# Inflammation contributes to trauma-induced coagulopathy by oxidation of multiple clotting factors

**DOI:** 10.1016/j.redox.2025.103956

**Published:** 2025-11-30

**Authors:** Chang Yeop Han, Alexander E. St John, Jung Heon Kim, Xu Wang, Kristyn M. Ringgold, Lauren E. Neidig, Ronald Berenson, Susan A. Stern, Nathan J. White

**Affiliations:** a Department of Emergency Medicine and Resuscitation Engineering Science Unit, Harborview Medical Center, University of Washington, Seattle, WA, USA; b Department of Comparative Medicine, University of Washington, Seattle, WA, USA; c Aequus Biopharma, Seattle, WA, USA; d Bloodworks Northwest Research Institute, Seattle, WA, USA; e Department of Emergency Medicine, Ajou University School of Medicine, Suwon, Republic of Korea

**Keywords:** Coagulopathy, Inflammation, Coagulation factor, Reactive oxygen species, Oxidation, Trauma-induced coagulopathy

## Abstract

Trauma-induced coagulopathy (TIC) induces anticoagulation and increases bleeding mortality. Inflammation and oxidative stress play an unknown role in TIC. We examined plasma from injured human trauma patients presenting to the Emergency Department compared to healthy controls to elucidate the contribution of inflammation and oxidative stress to anticoagulation during TIC. Trauma patients demonstrated coagulopathy by prolongation of clotting time assays and decreased thrombin generation in addition to increased pro-inflammatory cytokines and increased markers of oxidative stress. Clotting factors seven (FVII), ten (FX), and twelve (FXII) were oxidatively modified without quantitative changes, displaying decreased activity after trauma. Factor five (FV) was decreased in concentration and retained normal activity. Factor eight (FVIII) concentration and activity were increased after trauma. Clotting factor oxidation after exposure to activated human leukocytes *in vitro* also impaired thrombin generation and reproduced the oxidative and functional changes seen in trauma patients. Both antioxidant and anti-inflammatory treatments prevented clotting factor oxidation and TIC after trauma *in vivo* using a rodent TIC model. These results suggest that inflammation and oxidative stress contribute directly to anticoagulation during TIC by direct and selective oxidation of clotting factors. FXII may make a novel contribution to the pathophysiology of TIC by its oxidation.

## Highlights

• Blood clotting factors seven (FVII), ten (FX), and twelve (FXII) are oxidatively modified and dysfunctional with trauma-induced coagulopathy.
• Plasma inflammation and oxidation are associated with impaired thrombin generation after trauma.
• Antioxidant and anti-inflammatory treatments prevent oxidation and trauma-induced coagulopathy both in vitro and in vivo.

## Graphical abstract

**Figure:**
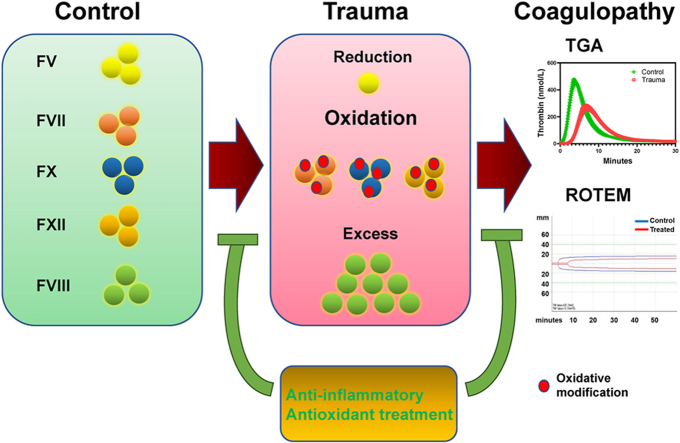

## 1 Introduction

Trauma induced coagulopathy (TIC) is an acute unbalancing of coagulation homeostasis after severe injury characterized by early hypocoagulable responses associated with increased bleeding followed by hypercoagulable responses with increased thrombosis [1]. Bleeding mortality within the first hours of injury is greatly increased in the presence of TIC [[2], [3], [4], [5], [6]].

The early hypocoagulable response in TIC has been described as impairment of thrombin generation, activation of hyperfibrinolysis (HF), and clotting factor consumption [[7], [8], [9]]. Low clotting factor activity in factors five (FV), seven (FVII), and ten (FX), presumably from consumption, have been associated with decreased thrombin generation after trauma [[9], [10], [11], [12]]. HF is mainly attributed to increased plasmin generation triggered by a release of tissue plasminogen activator (tPA) and activated protein C's inhibitory effect on plasminogen activator inhibitor 1 (PAI-1) [8,13,14].

Inflammation is also an important event after trauma, with the overall magnitude and time to resolution of the inflammatory response being associated with complications in trauma patients requiring intensive care [15]. This acute response arises from endothelial cell and peripheral leukocytes release of pro-inflammatory cytokines, chemokines, and reactive oxygen species (ROS) [16,17] generated by myeloperoxidases (MPO), NADPH oxidase (NOX), and mitochondria [[18], [19], [20], [21], [22], [23]]. We have found that oxidants generated from leukocytes after pro-inflammatory cytokine stimulation, and after trauma, are capable of directly oxidizing fibrinogen and impairing its polymerization, thus providing a novel and direct link between trauma, inflammation, and coagulopathy [17]. However, the overall susceptibility of clotting factors to oxidation and the overall impact of oxidation on coagulation system function in human trauma patients remains unknown.

The goal of this study was to examine the contribution of inflammation and clotting factor oxidation on the coagulation system in a cohort of human trauma patients by describing cytokine responses, thrombin generation and plasmin generation, clotting factor activity, concentration, and oxidation state. We also used *in vitro* methods to study potential mechanisms and therapeutic targets. Using thrombin generation (TGA) and plasmin generation assays (PGA), we found that thrombin generation was severely impaired while plasmin generation was intact in our cohort of Emergency Department trauma patients compared to normal healthy subjects. The extent of impairment of thrombin generation was associated with the presence of coagulopathy which was primarily associated with oxidation and dysfunction of FVII, FX and FXII, that could be rescued by increased FVIII concentration. Using an *in vitro* human leukocyte model and an *in vivo* rat trauma model, we found that both antioxidants and drugs blocking leukocyte inflammation could restore TGA to normal after polytrauma. This study challenges previous paradigms by establishing oxidative coagulopathy as a component of TIC with wide ranging effects across the coagulation system, offers new insight into its mechanisms, and offers new potential therapeutic targets.

## 2 Results

### 2.1 Trauma induces coagulopathy and increases inflammation

We enrolled 21 trauma patients admitted to the Emergency Department arriving within 3 h of injury having a broad range of injury severity scores (ISS) from 1 to 45 and compared them to 10 normal healthy donors (Supplemental Table 1). Average ISS was severe at 18.4, international normalized ratio (INR) was 1.16, revised trauma score (RTS) was 5.0, mechanism of blunt trauma was 52 %, and mortality was 14 % in this cohort (Supplemental Table 1).

**Fig. 1:**
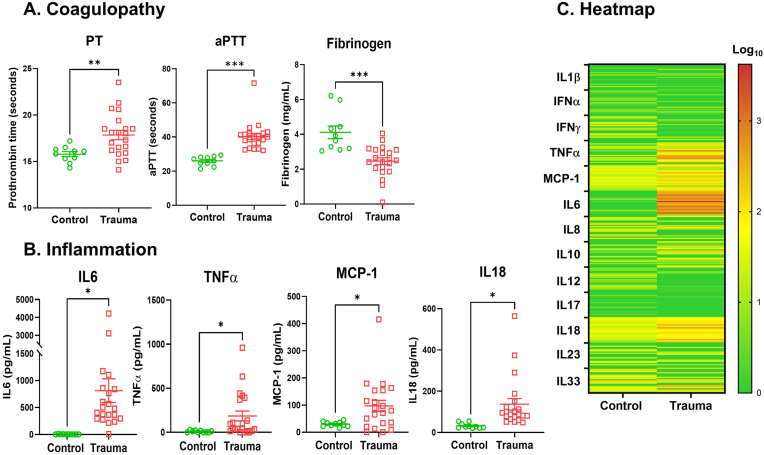
**Trauma induces inflammation, resulting in coagulopathy in humans.** Human plasma was collected from trauma patients (n = 21) and healthy controls (n = 10). **A**. Prothrombin time (PT) activated partial thromboplastin time (aPTT), and fibrinogen were measured using Ceveron alpha (mean ± standard error of the mean (SEM)). ∗∗p < 0.01, ∗∗∗p < 0.001, ∗∗∗∗p < 0.0005 vs. control. PT and aPTT panels (Mann-Whitney *U* test), Fibrinogen panel (Student's t-test). **B.** Cytokines were measured from plasma by multiplex cytokines (IL1β, IFNα, IFNγ, IL6, IL8, IL10, IL12, IL17, IL18, IL23, IL33, MCP-1, and TNFα) analysis kit. ∗p < 0.05 vs. control. Mann-Whitney *U* test. **C.** Heatmap of individual cytokines were plotted by the logarithm with base of 10. Scale bar with color change of green to red shows on right panel.

When compared to the control group, the trauma group's mean prothrombin time (PT), representing the extrinsic coagulation pathway, was increased (15.78 ± 0.27 vs. 17.86 ± 0.48 s, means ± SEM), mean activated partial thromboplastin time (aPTT), representing the intrinsic coagulation pathway, was increased (26.05 ± 0.87 vs. 40.01.21 ± 1.76 s), and fibrinogen was decreased (4.12 ± 0.19 vs. 2.46 ± 0.36 g/L) (Fig. 1A). Pro-inflammatory cytokines including IL6, TNFα, MCP-1, and IL18 were significantly increased with trauma (Fig. 1B and C). P-selectin and sCD40L, soluble plasma markers for endothelial cell damage were increased in trauma patients (Supplemental Fig. 1). Coagulation parameters were also very strongly correlated with pro-inflammatory cytokines, IL6, TNFα, MCP-1, and IL18 (Supplemental Fig. 2). However, correlation between coagulation parameters and soluble plasma markers for endothelial cell damage were either very weak or nonexistent (Data not shown). These results imply that inflammation caused by trauma contributed to TIC during the early stages of trauma in this cohort.

### 2.2 Thrombin and plasmin generation after trauma

**Fig. 2:**
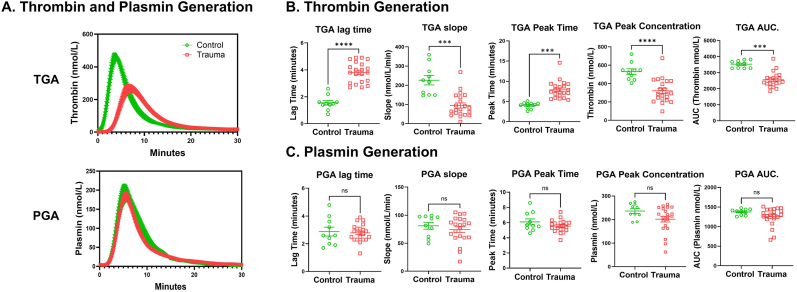
**Trauma causes the impairment of thrombin generation but stays intact plasmin generation in humans.** Plasma from trauma patients (n = 21) and healthy controls (n = 10) were subjected to analysis of TGA and PGA using Ceveron alpha as described in Methods. **A.** Cure of thrombin generation or plasmin generation from trauma patients (red) were plotted against normal controls (green). **B** and **C**, Various variables, lag time, slope, peak time, peak concentration and AUC. from TGA or PGA were plotted healthy controls; versus trauma patients with mean ± SEM. ns = not significant. ∗∗∗p < 0.001, ∗∗∗∗p < 0.0001 vs. control. TGA slope, peak time and AUC panels, PGA peak concentration and AUC panels (Mann-Whitney *U* test), others (Student's t-test).

Thrombin generation curves were severely decreased in the trauma group and were characterized by a rightward shift, decreased peak, and reduced AUC compared to controls (Fig. 2A and B). In contrast, plasmin generation curves were not different for the trauma group compared to control (Fig. 2A and C). TGA parameters were strongly associated with coagulation measurements and pro-inflammatory cytokines, while PGA showed very weak or no significant associations (Supplemental Figures 3, 4 and 5). Only peak concentration in PGA was negatively correlated with IL6, TNFα and IL18 while other parameters in PGA showed no relationship with cytokines (Supplemental Fig. 5B). Overall, these data also imply that TGA's were impaired relative to plasmin generation early after trauma, and increasing inflammation is associated with dysfunctional thrombin generation.

### 2.3 FV, FVII and FXII show dysfunction while FVIII is increased in trauma patients

**Fig. 3:**
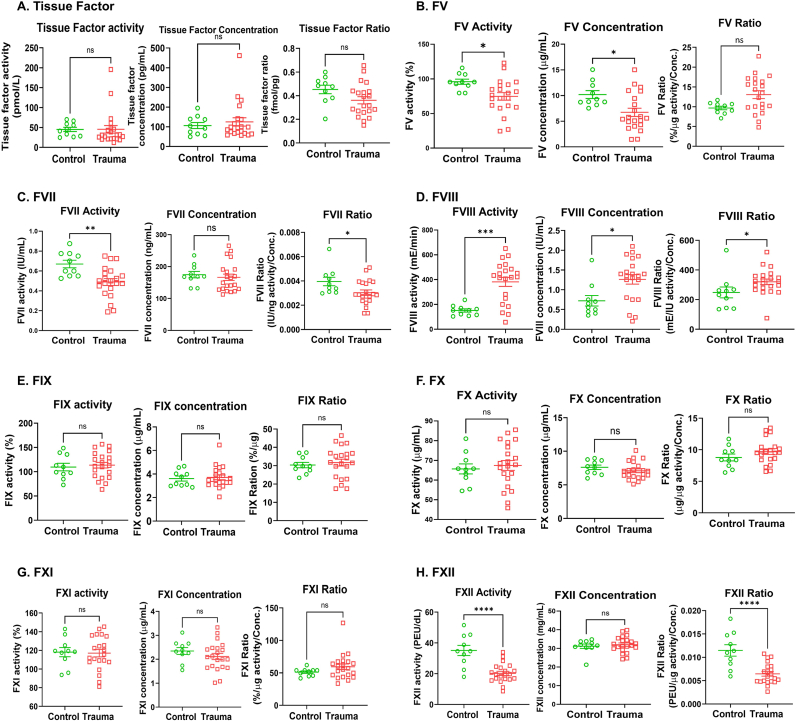
**Trauma shows unique features in clotting factors in humans.** The activity and protein concentration of each clotting factor, FV (**A**), FVII (**B**), FVIII (**C**), FIX (**D**), FX (**E**), FXI (**F**) and FXII (**G**) was measured using human plasma from trauma patients (n = 22) and healthy controls (n = 10) as described in Methods. Ratio of each clotting factor was calculated by dividing the activity of each factor with protein concentration. Data represent mean ± SEM. ns = not significant. ∗p < 0.05, ∗∗p < 0.01, ∗∗∗p < 0.001, ∗∗∗∗p < 0.0001 vs. control. FV ratio, FVII concentration, FVIII activity, FIX concentration, FXI ratio and FXII ratio panels (Mann-Whitney *U* test), others (Student's t-test).

We next assessed for factor dysfunction by measuring the concentration and activity of each clotting factor and calculating the activity to concentration ratio. FXII activity was decreased in trauma patients compared with normal controls while its protein concentration was unchanged (Fig. 3G). FXII ratio, representing individual activity per protein unit, was lower in trauma patients (Fig. 3G). FVII activity was reduced but protein concentration was normal yielding a decreased activity per protein unit ratio compared to controls (Fig. 3B). Tissue factor (FIII), FXI and FIX activity, concentration, and ratio were not different for trauma patients compared to controls (Fig. 3A–D and F). Similarly, FX activity, concentration, and ratio were not different from controls (Fig. 3E). Surprisingly, FVIII activity, concentration, and ratio were each substantially increased in trauma patients compared to controls (Fig. 3C). Finally, FV activity and concentration were decreased, and its ratio was unchanged for trauma patients compared to controls (Fig. 3A). FV, FVII and FXII activity showed strong associations with PT, aPTT, fibrinogen, and IL6 (Supplemental Fig. 6). FV, FVII and FXII activities correlated positively with TGA's (peak time and peak concentration), but did not correlate with PGA's (Supplemental Fig. 7). FIII, FVIII, FX and FXI activity had weak or no association with PT, aPTT, fibrinogen, TGA or PGA (Supplemental Figs. 6 and 7). FIII activity showed strong correlation with IL6 (Supplemental Fig. 6). Overall, these results indicate that early TIC is associated with a unique clotting protein signature of decreased FV, dysfunction of FVII and FXII, and increased FVIII, and imply that inflammation causes these unique changes of FV, FVII and FXII, leading to impaired thrombin generation, and consequently disrupting critical hemostatic balance towards anticoagulation.

### 2.4 Oxidative stress is high in trauma patients

**Fig. 4:**
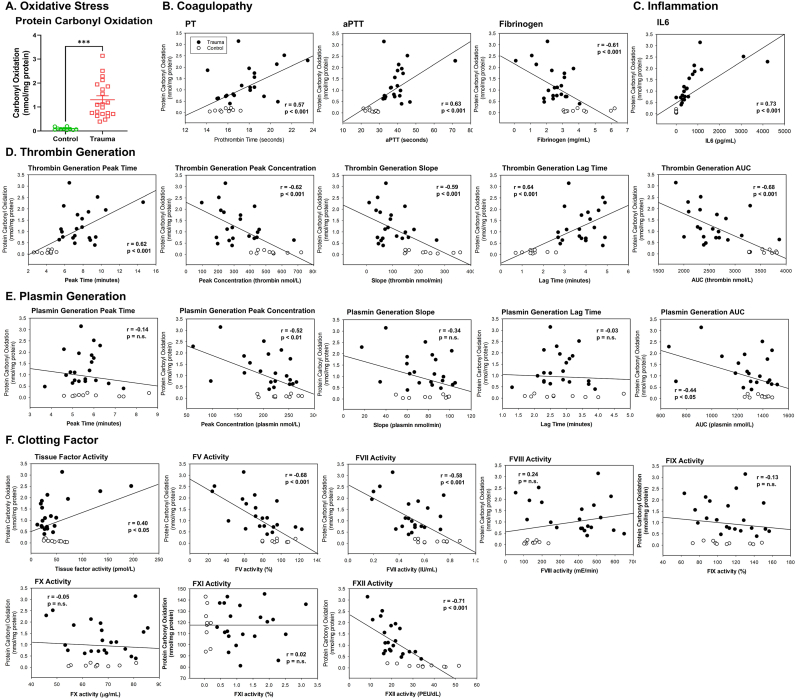
**Oxidative stress is increased and correlates with coagulopathy, inflammation, thrombin generation, and FV, FVII and FXII activities in trauma patients. A**. Oxidative stress was analyzed by measuring oxidative carbonyl modification in trauma patients (n = 21, closed circle) and healthy controls (n = 10, open circle) as described in Methods. Linear regression relationship was shown between oxidative stress versus coagulopathy (PT. aPTT and fibrinogen, **B**), IL6 (**C**), TGA (peak time, peak concentration, slope, lag time and AUC., **D**), PGA (peak time, peak concentration, slope, lag time and AUC., **E**) or the activity of each clotting factor (**F**) using Pearson correlation coefficient. Data represent mean ± SEM. ∗∗∗p < 0.001 vs. control. Mann-Whitney *U* test.

To identify the cause of clotting factor dysfunction as measured by decreased FVII and FXII activity ratios, we evaluated the extent of oxidation of clotting factors in trauma patients. We first found that overall oxidative carbonyl protein modifications were increased in the plasma of trauma patients compared to controls (Fig. 4A). To confirm an increase of the oxidative stress in trauma patients using an additional method other than ELISA, we performed multiplex fluorescent Western blot for oxidative carbonyl modification using OxyBlot (red band) and human albumin specific antibodies (green band) from immunoprecipitated albumin of trauma patient and control plasmas (Supplemental Fig. 8). We found that the most abundant protein in plasma, albumin, as well was oxidatively modified in trauma patients. In addition, the extent of oxidative modification was strongly associated with coagulation parameters (PT, aPTT and fibrinogen concentration) and the level of pro-inflammatory cytokine IL6 (Fig. 4B and C). All TGA variables including lag time, peak time, peak concentration, slope, and AUC were highly correlated with plasma protein oxidation, while PGA's were not correlated (Fig. 4D and E). When examining individual clotting factor activities, we found that FV, FVII, and FXII activities were strongly and negatively correlated with plasma protein oxidative modification (Fig. 4F). These findings imply that inflammation causes oxidative modifications that link to impaired thrombin generation and contribute to coagulopathy in trauma patients.

### 2.5 FVII, FX and FXII are oxidized in trauma patients and by activated leukocytes

**Fig. 5:**
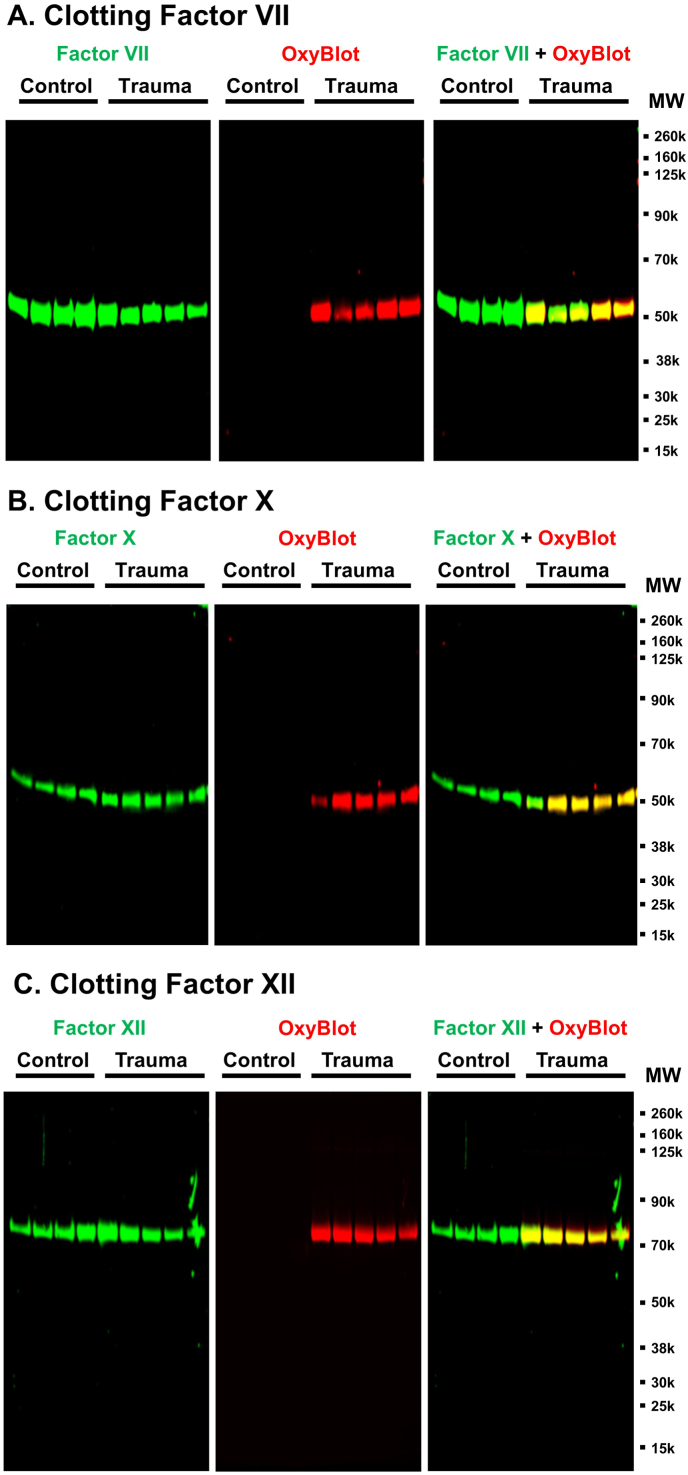
**FVII, FX and FXII are oxidized in trauma patients.** FV (**A**), FX (**B**) and FXII (**C**) were immunoprecipitated using monoclonal antibodies against FV, FX and FXII from plasma of trauma patient (n = 5) and healthy controls (n = 4) as described in Methods. Purified FV, FX and FXII was analyzed by OxyBlot and immunoblotting using FV, FX or FXII – specific antibodies. Representative blots are displayed.

**Fig. 6:**
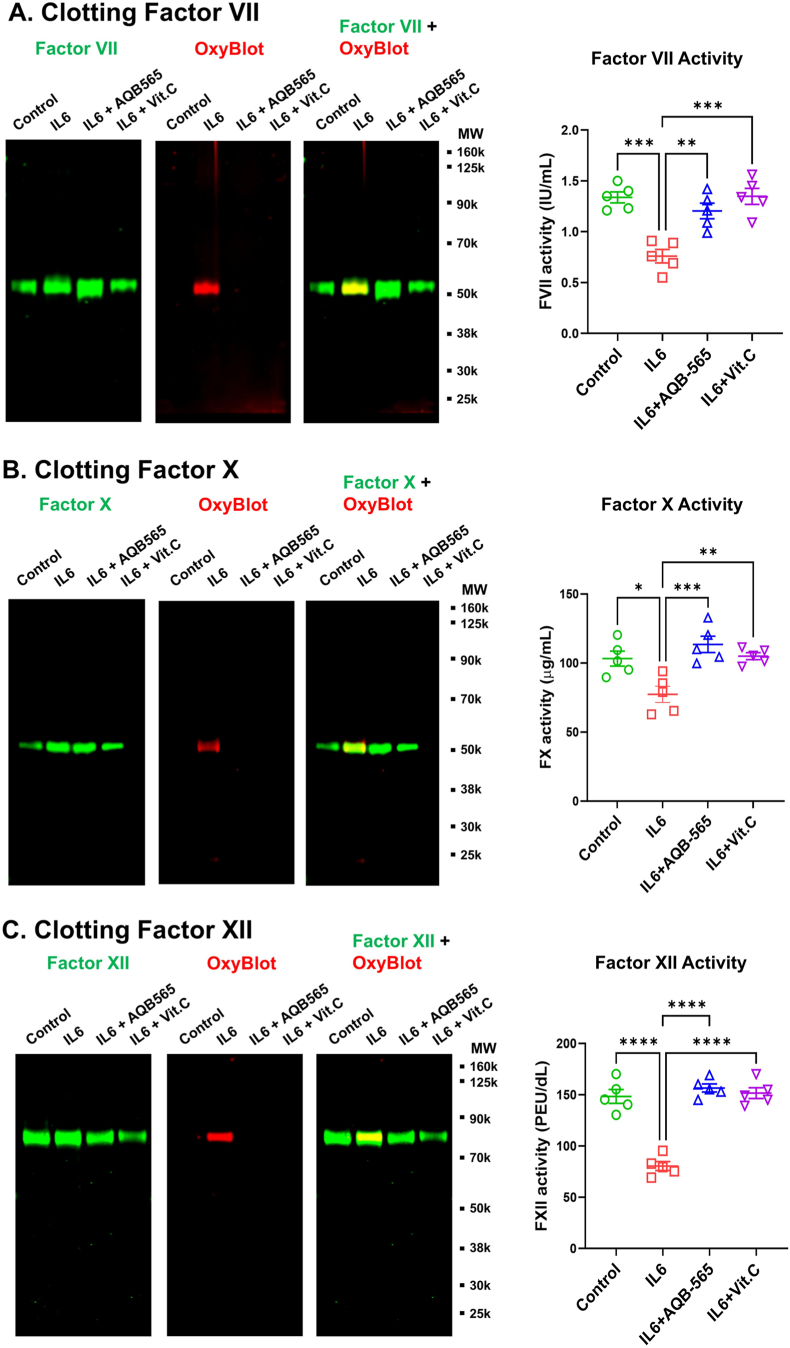
**IL6-activated leukocytes mimic oxidation and reduce the activity of clotting factors shown in trauma patients while the anti-inflammatory protein, AQB-565 and antioxidant, vitamin C prevent these phenomena.** IL6-stimulated RBC-lysed leukocytes from healthy donors (n = 5) were incubated with FVII, FX and FXII, or AQB-565 and vitamin C as described in Methods for 4 h. After that, FVII, FX and FXII were isolated by immunoprecipitation as described in Methods. Purified FV (**A**), FX (**B**) and FXII (**C**) was analyzed by OxyBlot and immunoblotting using FV, FX or FXII – specific antibodies (left panel). Representative blots are displayed. Also, each activity of these coagulation factors was measured in isolated FVII, FX and FXII as described in methods (right panel). Data represent mean ± SEM. ∗p < 0.05, ∗∗p < 0.01, ∗∗∗p < 0.001, ∗∗∗∗p < 0.0001 vs. IL6. ANOVA and Tukey post-hoc test.

Since only the activities of FVII and FXII were decreased without changes of protein concentration and their oxidative modification were strongly and negatively related with the activities of FVII and FXII in trauma patients, we attempted to detect direct oxidation of these proteins after trauma. We excluded FV because its reduced protein concentration likely accounts for its reduced activity and association with coagulation parameters, inflammation, plasma oxidation and thrombin generation. Moreover, FV retained normal activity after trauma, suggesting a quantitative rather than qualitative change. In addition, since FX represents common coagulation pathway activation, and is the most abundant protein amongst other clotting factors, we also examined oxidative modification in FX. To determine if clotting factors were individually oxidized, we next immunoprecipitated FVII, FX, and FXII from trauma patient and control plasmas, and performed multiplex fluorescent Western blot for oxidative carbonyl modification using OxyBlot (red band) and factor-specific antibodies (green band) (Fig. 5). We found that FVII, FX and FXII were oxidized in trauma patients, but not in normal controls. Purified clotting factors were then incubated *in vitro* with IL6-stimulated leukocytes using an established *in vitro* model with and without the presence of the antioxidant, vitamin C or an anti-inflammatory melanocortin-derived fusion protein, AQB-565, previously demonstrated to block fibrinogen oxidation in the same model at a similar concentration [17]. We found that FVII, FX, and FXII were oxidized after exposure to activated leukocytes and their oxidation was prevented by vitamin C and AQB-565 (Fig. 6). Clotting factor activity was also decreased for each factor upon its oxidation following activated leukocyte exposure, and its activity was retained in the presence of vitamin C and AQB-565 treatments (Fig. 6). These results indicate that oxidative stress generated by activated leukocytes can directly modify multiple important clotting factors.

Interestingly, we found that FX was also oxidized in trauma patients but did not demonstrate a similar loss of activity when compared to FVII and FXII oxidation in trauma patients. To investigate this further, we first demonstrated that FX oxidation decreases its activity *in vitro*. We then co-incubated leukocyte-oxidized FX with FVIII which acts as a co-factor for FIX to activate FX and was noted to be increased in the trauma cohort. We found that excess FVIII could compensate for and restore lost FX activity induced by its oxidation (Supplemental Fig. 9). These data imply that oxidation of FVII, FX and FXII blunts their activities. However, excess FVIII can compensate for the decreased activity of oxidized FX.

### 2.6 Importance of individual clotting factor oxidation to overall clot formation using factor-deficient plasma

**Fig. 7:**
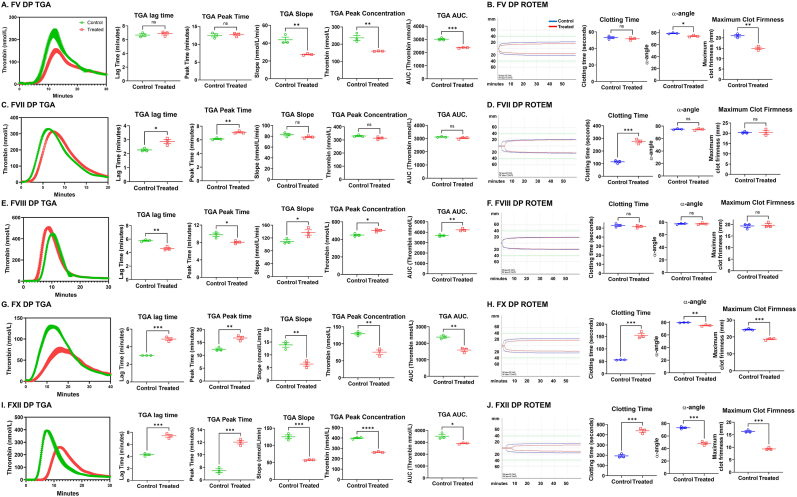
**Each clotting factor shows distinct and unique roles in thrombin generation and ROTEM.** TGA (**A**, **C**, **E**, **G**, and **I**) and ROTEM (**B**, **D**, **F**, **H**, **J**) were performed using each clotting factor-specific DP (n = 3) as described in Methods. **A** and **B**. control amount (control) of FV which we found in healthy controls or reduced amount (treated) of FV which we found in trauma patients were added in FV DP. Variables from TGA, lag time, peak time, slope, peak concentration and AUC, or variables from ROTEM, clotting time, a-angle, maximum clot firmness were obtained in each experiment as described in Methods. **C** and **D**. control FVII (control) and oxidized FVII (treated) which were obtained by incubation with leukocytes as described in Methods were added to FVII DP. **E** and **F**. control amount (control) or excess amount (treated)of FVIII were added to FVIII DP. **G** and **H**. control FX (control) and oxidized FX (treated) which were obtained by incubation with leukocytes as described in Methods were added to FX DP. **I** and **J**. control FXII (control) and oxidized FXII (treated) which were obtained by incubation with leukocytes as described in Methods were added to FXII DP. Data represent mean ± SEM. ∗p < 0.05, ∗∗p < 0.01, ∗∗∗p < 0.001, ∗∗∗∗p < 0.0001 vs. control. Student's t-test.

To examine the importance of individual and combined coagulation factor oxidation, we next investigated the individual effect of each coagulation factor on thrombin generation using TGA and overall plasma clot formation using rotational thromboelastometry (ROTEM). We used specific factor-deficient plasmas (DP) spiked with normal or oxidized clotting factors at the concentration which we found in trauma patients or normal controls (Fig. 7). Using FV-DP, a decreased FV concentration decreased TGA slope, peak concentration and AUC, but did not affect lag time and time to peak compared with normal controls (Fig. 7A). Similarly, using ROTEM, reduced FV concentration decreased α-angle and MCF, but did not affect CT (Fig. 7B). Using FVII-DP, adding oxidized FVII increased TGA lag time and time to peak, but did not affect slope, peak concentration, or AUC compared with controls (Fig. 7C). Again, using ROTEM, we found a significant increase in CT, but no changes of α-angle and MCF (Fig. 7D). Using FVIII-DP, excess FVIII shortened TGA lag time and peak time, increased slope, peak concentration, and AUC, while making no discernible changes in ROTEM parameters (Fig. 7E and F). Using FX-DP, adding oxidized FX increased TGA lag time and peak time, and decreased slope, peak concentration and AUC, and worsened all ROTEM parameters (Fig. 7G and H). Using FXII-DP, adding oxidized FXII substantially delayed TGA lag time and time to peak, and remarkably decreased slope, peak concentration and AUC, while also increasing CT and decreasing α-angle and MCF for ROTEM (Fig. 7I and J).

Experiments were then repeated with addition of excess FVIII to determine its effects on thrombin generation and ROTEM parameters in the presence of individual and combined clotting factor oxidation. FVIII restored peak concentration and AUC in TGA, and α-angle and MCF in ROTEM when added to FV-DP (Supplemental Fig. 10). In addition, adding an excess of FVIII restored TGA and ROTEM parameters for individually decreased FV, oxidized FVII, FX, and FXII, but excess FVIII failed to rescue TGA and ROTEM when all clotting factors defects were combined (Supplemental Fig. 10). To reproduce clotting factor behavior noted in the trauma patient cohort, we tested a combination of reduced FV, excess FVIII, and oxidized FVII, FX, and FXII. This combination induced a phenotype similar that seen in the trauma patient cohort with a delayed TGA lag time, prolonged time to peak, and decreased TGA slope, peak concentration and AUC (Supplemental Fig. 10I).

Summarized, these data imply that decreasing FV reduces fibrin clot stiffness, but not speed of clot formation. Oxidized FVII slows clot formation, and does not affect stiffness. Additionally, oxidized FX slows clot formation and decreases stiffness. Oxidized FXII also slows clot formation and decreases clot stiffness. Finally, excess FVIII can restore clot formation when single factor defects are present. However, excess FVIII failed to rescue clot formation when multiple clotting defects were present simultaneously.

### 2.7 Intervention study using rat polytrauma model

**Fig. 8:**
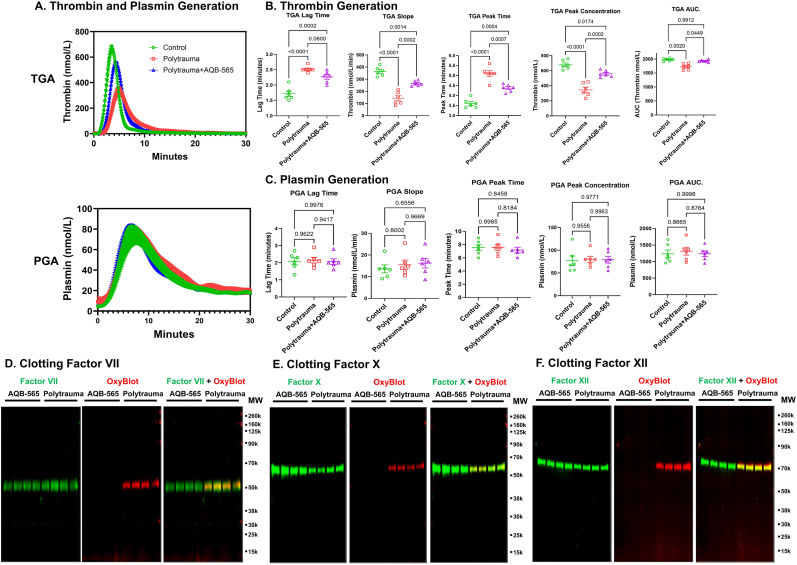
**Thrombin generation is impaired in polytrauma-induced rat while the anti-inflammatory protein, AQB-565 restores it.** Polytrauma-induced rats were injected with saline (control, n = 6) or AQB-565 (treatment, n = 6) as described in Methods. TGA and PGA were performed using plasma from control rats (n = 6), polytrauma-induced rats with saline and polytrauma-induced rats with AQB-565. **A**. Each curve in TGA and PGA was plotted. **B**. Variables in TGA, lag time, slope, peak time, peak concentration and AUC, were analyzed. **C**. Variables in PGA, lag time, slope, peak time, peak concentration and AUC, were analyzed. Data represent mean ± SEM. p value showing in each relevant comparison. TGA AUC panel (Kruskal-Wallis and Dunn post-hoc test), others (ANOVA and Tukey post-hoc test). Purified FV (**D**), FX (**E**) and FXII (**F**) of plasma from polytrauma-induced rats treated with saline (polytrauma) or AQB-565 (n = 4) was analyzed by OxyBlot and immunoblotting using FV, FX or FXII– specific antibodies. Representative blots are displayed.

We then investigated the effects of ani-inflammatory therapy on thrombin generation and clotting factor oxidation *in vivo* using an established rat TIC model [17]. First, we examined the protein concentration of anti-inflammatory melanocortin fusion protein AQB-565, administered at the same concentration previously seen to prevent fibrinogen oxidation, in rat plasma and found that it was substantially increased compared to saline-treated controls (Supplemental Fig. 11). Like results seen in trauma patients, combined trauma and hemorrhagic shock impaired thrombin generation without affecting plasmin generation (Fig. 8A). Moreover, the anti-inflammatory melanocortin fusion protein AQB-565, suppressed cytokine elevations and restored thrombin generation (Fig. 8A and B) when given shortly after onset of hemorrhagic shock. There were no significant changes in plasmin generation between control, polytrauma and polytrauma treated with AQB-565 (Fig. 8C). Also, polytrauma oxidatively modified FVII, FX and FXII, and their oxidation was prevented by AQB-565 treatment (Fig. 8D, E and F). This result implies that anti-inflammatory treatment targeting peripheral leukocytes via melanocortin receptors early during trauma can prevent factor oxidation.

## 3 Discussion

The main findings of this study were that multiple changes in clotting factor activity, concentration, and oxidation were present in Emergency Department trauma patients. These changes were associated with impaired thrombin generation. The initiators of the extrinsic, intrinsic, and common clotting pathways, FVII, FXII, and FX, respectively, were oxidized, became dysfunctional, and contributed to coagulopathy both *in vitro* and *in vivo*. These changes were also associated with FV consumption and could be partially reversed with increased FVIII concentration and activity. Finally, we demonstrated protection against clotting factor oxidation using antioxidant and anti-inflammatory treatments *in vitro* and *in vivo,* establishing oxidative coagulopathy as an important novel mechanistic component of TIC and a therapeutic target.

Thrombin generation was significantly decreased while plasmin generation was not affected in our trauma cohort compared to healthy controls. These findings are consistent with other work demonstrating a relative decrease in thrombin generation in trauma patients with TIC suggesting the presence of upstream inhibition of clotting factor concentration or activity [13]. Alternatively, we did not find an associated increase of plasmin generation. This finding conflicts with established literature demonstrating fibrinolytic activation by increased plasmin-antiplasmin complex, shortened euglobulin clot lysis time, and hyperfibrinolysis using viscoelastic hemostatic assays during TIC that has been attributed to increased tPA release or disinhibition of serpins by the activated protein C pathway [[24], [25], [26]]. While there is limited previous work examining plasmin generation assays directly, Lawson et al., noted that trauma patients identified as having hyperfibrinolysis also curiously did not also exhibit increased plasmin generation in response to tPA [27]. It is possible that fibrin clot structure or alternative lytic pathways are responsible. We also suspect that our limited trauma cohort may not have been injured severely enough to elucidate overt hyperfibrinolysis, which is quite rare, presenting in only 5 % of severely injured trauma patients with TIC. Therefore, our cohort described an overall state of deficient thrombin generation relative to retained plasmin generation, suggesting the presence of upstream clotting factor deficiency, dysfunction, or inhibition.

Thrombin generation is generally governed by the integrity of the upstream clotting factor cascade supported by available surfaces for assembly of factor complexes to support clot initiation and propagation. The intrinsic and extrinsic activation pathways provide redundant mechanisms for initiation of thrombin generation via the common pathway. The intrinsic pathway is initiated by activation of FXII on negatively charged surfaces such as anionic phospholipid and collagen, leading to FXI activation along with kallikrein and high molecular weight kininogen [28]. FXI activates FIX and promotes the common pathway by FVIII, which together with FV, act as cofactors for FX and subsequent conversion of prothrombin to thrombin. Alternatively, the extrinsic pathway generates thrombin by the common pathway after its initiation by tissue factor via FVII activation. We found that the key initiators of the intrinsic, extrinsic, and common coagulation pathways, FXII, FVII, and FX, respectively were each oxidized after trauma. Oxidation decreased their activities and correlated with decreased thrombin generation and poor clot formation [17]. Oxidative dysfunction of single clotting factors produced distinct but limited effects on thrombin generation, while combined oxidative dysfunction of multiple of these key initiators, as seen in our trauma cohort, produced global thrombin generation deficits and poor clot formation that could not be restored with excess FVIII *in vitro*, suggesting a synergistic contribution of multiple clotting factor oxidation to TIC.

Several clotting factors have demonstrated similar susceptibility to oxidative and nitrosative modification by free radical and oxidative stressors. Fibrinogen contains multiple amino acids that are susceptible to oxidative modification, producing a range of functional outcomes [29]. A review of plasma protein carbonylation by Madian et al., noted evidence for coagulation protein carbonylation in healthy individuals, most notably fibrinogen, as natural targets of ROS, but did not find similar oxidation of other clotting factors in the healthy state [30]. Fibrinogen oxidation affecting fibrin clot formation is also a hallmark of cardiovascular inflammation [31]. Notably, these changes were reversible with IL6 blockade, highlighting the clinical significance of fibrinogen oxidation in inflammatory vascular disease. More specifically to trauma, we previously found that oxidation of the fibrinogen A-αC domain via methionine sulfoxide formation inhibited lateral protofibril aggregation and induced softer clot formation in plasma sampled from trauma patients with TIC [32]. More recently, we found that fibrinogen was capable of being oxidized directly by activated neutrophils and monocytes through a pathway involving myeloperoxidase and mitochondrial superoxide formation, suggesting a direct role for leukocyte-specific inflammation as an initiating stimulus [16,17]. The current work adds to this evidence by demonstrating that FVII, FXII, and FX are also directly oxidized towards dysfunction in the presence of activated human leukocytes. Similarly, sodium hypochlorite and chloramines, produced by activated leukocytes, can irreversibly oxidize FV, FVIII, and FX and inhibit platelet aggregation, suggesting potential wide-ranging effects of leukocyte-induced oxidation on clot formation and hemostasis [33]. In addition, oxidation of recombinant activated clotting factor VII (FVIIa) by hydrogen peroxide selectively modifies methionine residues (Met298 and Met306), weakening its binding to soluble tissue factor and reducing its catalytic activity in complex with tissue factor [34]. FXIII is also susceptible to nitrosylation by nitric oxide by its S-nitrosylation of a highly reactive cysteine residue [35]. Of note, mechanistic investigations describing the culprit oxidative modifications and their effects on FXII and FX function are currently lacking in the literature. However, we previously showed that ROS from neutrophils and monocytes in sterile inflammation and polytrauma rat models caused oxidative modifications in fibrinogen [16,17]. Therefore, the same mechanisms of oxidation of FVII, FX and FXII may be at work. In future studies, specific deficiency of leukocytes using knockout models or treatment of clodronate which kill myeloid cells would clarify the role or peripheral leukocyte contribution to TIC after trauma. Other future studies are needed to identify the specific oxidative modification sites and the exact form of modification on coagulation proteins using mass-spectrometry.

Several interesting quantitative changes in clotting factor concentrations were also noted in our study. Namely, FV was significantly decreased after trauma while retaining normal activity. This finding agrees with rapid consumption of FV as a hallmark of TIC, where its deficiency was previously observed in all patients with TIC and correlated with impaired thrombin generation according to Rizoli et al. [9]. Increased activation of the protein C system during trauma with preferential proteolysis of FV has been proposed as a mechanism for its rapid loss during TIC [11]. Interestingly, we also found a significant *increase* of FVIII concentration with retained activity in our trauma patient cohort compared to healthy controls. This result is in contrast with studies reporting reduced FVIII activity during TIC describing a phenotype of global factor consumption [11,36]. However, several studies have reported that FVIII concentration is unaffected or increased in trauma cohorts when blood is collected acutely at hospital arrival. Burggraf et al., reported FVIII as being the sole clotting factor with increased activity after severe trauma when measured at hospital arrival [10]. In addition, FVIII was the only clotting factor that did not decrease significantly with increasing degree of hemorrhagic shock as measured by base deficit after trauma [37]. Importantly, we further identified FVIII as being quantitatively increased in concentration after trauma, suggesting acute synthesis or more likely acute release from storage depots by active secretion from various cells, as FVIII is known to be produced and stored by a wide range of endothelial cell types [38]. The physiological implications of increased FVIII concentration after trauma are important, because we found using factor DP that isolated oxidative dysfunction of FVII, FXII, and importantly FX, could be mitigated in the presence of excess of FVIII. Therefore, FVIII released into the bloodstream after trauma may represent an adaptive response intended to counteract inflammation-induced coagulopathy. We attribute the reason why FX activity was maintained in the plasma after trauma in our cohort to the increased concentration of FVIII. Although, it is also important to recognize that when spiking FVIII concentrations like that measured in our trauma cohort, we could not fully rescue thrombin generation when multiple clotting proteins were simultaneously oxidized.

We found a decreased activity of FVII after trauma resulting in an apparent “oxidative dysfunction” despite no changes in protein concentration. Congenital FVII deficiency is a relatively common bleeding disorder but FVII deficiency can also be rarely acquired during various illnesses and is typically associated with cancer and inflammation [39]. The use of recombinant activated clotting factor VIIa (FVIIa) for bleeding trauma patients has been studied and is associated with improved survival in retrospective studies, while a prospective randomized controlled trial for refractory bleeding after trauma demonstrated a reduction of blood transfusion need without mortality benefit, making it rarely used as single agent today [40,41].

Notably, FXII also experienced oxidative dysfunction that was strongly associated with inflammation, clot initiation, and clot formation. FXII initiates the intrinsic clotting cascade by its activation on negatively charged surfaces but is not required for hemostasis. Clinical and animal studies of isolated FXII-deficient mice and humans consistently show that FXII is dispensable for physiological clot formation, as its absence does not lead to increased spontaneous or trauma-associated bleeding [42]. This concept is based on the fact that other clotting factors can co-operatively work to promote hemostatic sufficiency in the absence of FXII [43]; i.e. in intact clotting cascade, amplification system including FVIII, FIX and FX would magnify the signal and make more than enough thrombin in lieu of FXII. Our results challenge this dogma by demonstrating that oxidative FXII dysfunction is present during TIC and contributes quite significantly to abnormal thrombin generation and clot formation. Other work does support a role for intact FXII playing an important role for promoting thrombus formation during high blood flow rates at wounds, and augmenting hemostasis after trauma, especially in the case of exposure to activating surfaces [44,45].

Our results suggest several potential therapeutic approaches for both prevention and treatment of TIC. Preventing oxidation and inflammation using either antioxidants or anti-inflammatory approaches could independently reduce clotting factor oxidation and TIC both *in vitro* and *in vivo*. We found that the anti-inflammatory protein AQB-565 blocked clotting factor oxidation and restored their activities when given at the onset of fluid resuscitation during treatment of hemorrhagic shock in a rat trauma model. We have also shown that N-acetylcysteine administration in trauma can reduce the hemostatic protein von Willebrand factor and improve organ perfusion [46]. Antioxidant cocktails are currently used to reduce organ failure and mortality for critically ill trauma patients [47,48]. However, antioxidant supplementation is started at hospital or ICU admission, far beyond the therapeutic window required for prevention or treatment of TIC. Similarly, randomized trials of combination of anti-inflammatory and antioxidant cocktails for treatment of sepsis have been criticized for lack of efficacy due to their initiation late in the course of sepsis treatment, typically only after vasopressors have been initiated [[49], [50], [51]]. Given the narrow therapeutic window that exists for treatment of TIC, a similar approach to tranexamic acid therapy, where treatment is required within the first hours of injury, may be required [52].

In parallel, FVIII could hold therapeutic promise. An excess of FVIII partially compensated for reduction of FV and oxidation of FVII, FX and FXII using factor DP *in vitro*. These results suggest several potential therapeutic approaches for both prevention of TIC and its treatment using specific factor replacement strategies. For example, retrospective studies of concentrated clotting factors in the form of prothrombin complex concentrates (PCC) of various compositions have noted an association with reduced blood product transfusion requirements and rapid normalization of INR in trauma patients with coagulopathy [53]. However, the PROCOAG trial of empiric 4- factor PCC (which does not contain FVIII) for trauma patients at risk of massive transfusion demonstrated no reduction of blood products transfused and an increased risk of thrombosis [54]. Our results demonstrate an emergent effect of excess FVIII on thrombin generation and clot formation by its support of FX activity and suggest that a more precise approach to factor replacement using FVIII may be useful for treatment of TIC.

In summary, we present a novel role for inflammation and oxidation of FVII, FXII, and FX in the pathophysiology of TIC. Notably, there was a strong influence of FXII oxidation on thrombin generation and clot formation that has not been previously appreciated. In addition, excess FVIII could restore the effects of single clotting factor dysfunction on thrombin generation, likely by its effects on FX. Antioxidant and anti-inflammatory therapy could prevent oxidative coagulopathy and may provide promising approaches towards treatment of clinical TIC. Further investigation is required to identify specific oxidative modifications associated with clotting factor dysfunction and the potential utility of FVIII, antioxidants, and anti-inflammatory treatments as novel therapeutics.

## 4 Methods

### 4.1 Human subjects

This study was approved by the University of Washington Human Subjects Division Institutional Review Board and included adult trauma patients aged 18 years or older, who met criteria for full trauma team activation and severe injury at Harborview Medical Center, Seattle, Washington, USA. We excluded trauma patients who arrived at the Emergency Department more than 3 h after injury, received more than 2 L of crystalloid fluid, more than 1 unit of whole blood, or 2 units of component blood products of any kind prior to blood sampling. We also excluded patients having burns of more than 5 % total body surface area, who received tranexamic acid prior to initial blood draw, who were known to have congenital coagulopathy or taking anticoagulants or antiplatelet medication, or who were pregnant, prisoners or transferred from another facility. Normal healthy adult volunteers were recruited at the Bloodworks Northwest Research Institute, Seattle, Washington to serve as controls. Whole blood samples were collected in trisodium citrate and centrifuged to platelet-poor plasma and stored at −80 °C for later analysis. Shock index was calculated as heart rate divided by systolic blood pressure and a shock index >0.7 represents increased risk of hemorrhagic shock [55].

### 4.2 Multiplex cytokine, prothrombin time (PT), activated partial thromboplastin time (aPTT) and fibrinogen measurements

Cytokines and chemokines (IL1β, IFNα, IFNγ, IL6, IL8, IL10, IL12, IL17, IL18, IL23, IL33, MCP-1, and TNFα) from human plasma were measured using the multiplex immunoassay LEGENDplex Human Inflammation Panel I (Biolegend) following the manufacturer's instructions. Soluble P-selectin and sCD40L from human plasms were measured using the customized multiplex immunoassay LEGENDplex (Biolegend) following the manufacturer's instructions. Samples were read using an Apogee flow cytometer and Histogram. v.6.0 (Apogee). Data analysis was performed with the LEGENDplex Data Analysis Software v.8 (Biolegend).

PT, aPTT and fibrinogen concentration from human plasma were measured using a fully automated photo-optical coagulation instrument, Ceveron-Alpha (Technoclone) according to manufacturer's instruction. Briefly, PT, aPTT and fibrinogen were measured with TechnoClot PT plus and Daptin and fibrinogen reagent kits (Technoclone), respectively.

### 4.3 Thrombin and plasmin generation assay

Thrombin generation assay (TGA) was performed with Ceveron TGA RC (Technoclone, #5006013) or RA (Technoclone, #5006205) kits using Ceveron-Alpha (Technoclone) according to manufacturer's instruction.

Plasmin generation assay (PGA) was also performed using Ceveron-Alpha (Technoclone) as described previously [56]. First, we calibrated plasmin concentration using human plasmin (Zedira, #P012, 1 μmol/L in 5 mg/mL bovine serum albumin, 5 mg/mL α-lactose monohydrate, 0.2 % acetate buffer, pH 4). We modified the trigger solution by adding recombinant tissue plasminogen activator (rtPA, Alteplase, Genentech, #NDC 50242, 0.5 pmol/L). Fluorescent PGA substrate (0.5 mmol/L Boc-Glu-Lys-AMC, Bachem, #4007739, 16.6 mmol/L CaCl2, 60 mg/mL bovine serum albumin in 20 mmol/L HEPES, pH 7.3) was used as a reporter of plasmin activity.

TGA and PGA generated various values to indicate the activities of thrombin and plasmin, respectively [57]: Lag time indicates the time-point when thrombin or plasmin are first generated. It corresponds to the time when the fibrin clot is formed (clotting time) or when fibrinogenolysis starts. Peak time indicates the time-point when thrombin or plasmin are maximum. Peak concentration indicates the maximum concentration of thrombin or plasmin. Slope indicates the velocity in which thrombin or plasmin are generated between the lag time and peak time. The area under the curve (AUC) indicates total concentration of thrombin or plasmin generation.

### 4.4 Clotting factor activity and concentration measurement

Individual clotting factor protein concentration and activity were measured from the plasma of trauma patients and healthy controls. Individual clotting factor protein concentration was measured by specific ELISA kits from Abcam (FIII, #ab220653, FV, #ab137976, FVII, #ab108830, FVIII, #ab272771, FIX, #ab108831, FX, #ab289650, FXI, #ab108834, FXII, #ab108835). The activity of FIII was measured using human tissue factor assay kit (Abcam, #108906). The activities of FV, FIX and FXI were measured with deficient plasma (DP) for FV (Techoclone, #5134004), FIX (Technoclone, #5164008), or FXI (Technoclone, #5184004) and DAPTTIN TC (Technoclone, #5035060) using Ceveron-Alpha (Technoclone) according to manufacturer's instruction. The activity of FVIII was measured by two step chromogenic assay with FVIII:2G Cal (Techoclone, #5344104) and FVIII:CH (Technoclone, #5322103) using Ceveron-Alpha (Technoclone) according to manufacturer's instruction. The activities of FV and FX were measured by FVII and FX human chromogenic activity kit (Abcam, #ab108830 and #ab108833). The activity of FXII was measured with FXII assay kit (Abcam, #ab241041). To identify clotting factor dysfunction, we calculated the ratio between activity and protein concentration for each clotting factor and compared these ratios between trauma patients and healthy controls.

### 4.5 Plasma protein oxidation measurement

Overall oxidation of plasma proteins were measured using OxiSelect protein carbonyl ELISA kit (Cell Biolabs, #STA-310) according to manufacturer's instruction. Whole plasma proteins were derivatized with 2,4-dinitrophenylhydrazone (DNP-hydrazone) to detect carbonyl formation, a standard and stable indicator of protein oxidation, and quantified by ELISA using anti-DNP antibody. Briefly described, carbonyl-oxidized BSA and reduced-form of BSA provided by manufacturer were serially diluted for preparing protein carbonyl BSA standard. After measuring protein concentration of plasma, 10 μg/mL of each plasma protein was plated in 96-well protein binding plate. After overnight incubation at 4 °C and washing, DNPH working solution was added and incubated for 45 min at room temperature in the dark. After extensively washing, blocking solution was incubated for 1 h at room temperature. After washing again, serial incubation with anti-DNP antibody and HRP conjugated secondary antibody respectively were performed for 1 h each. After developing the color using substrate solution, absorbance if each well was read at 450 nm using multiplate reader.

### 4.6 Immunoprecipitation of albumin, FVII, FX and FXII

To investigate the function or the extent of oxidative modification of albumin, FVII, FX and FXII in trauma patients, we purified albumin and these clotting factors from plasma of human patient or rat TIC model using immunoprecipitation. Briefly, mouse monoclonal antibodies for albumin (Abcam, 15C7, #AB10241), FVII (Invitrogen, AD-1, #MA5-17635), FX (Enzyme Research, 520, #Mab-FX) and FXII (Enzyme Research, 140, #Mab-FXII) in human samples, or rabbit poly clonal antibodies against FVII (Invitrogen, #PA5-115207), FX (Biorbyt, #orb556640), FXII (Proteintech, #12551-1-AP) were covalently immobilized onto Dynabead M − 270 epoxy using Dynabeads co-immunoprecipitation kit (Novex, #1431D) following manufacturer's instructions. These beads were incubated with human plasma from trauma patients and controls, or with plasma from polytrauma-induced rat treated with saline or AQB-565, washed and eluted according to manufacturer's instruction. Final eluted purified albumin, FVII, FX and FXII were dialyzed by PBS and quantified by ELISA.

### 4.7 OxyBlot and multiplex fluorescent Western blot analysis

To better quantify albumin, FVII, FX and FXII oxidative modifications, we performed multiplex fluorescent Western blot using mouse monoclonal antibodies against albumin, FVII, FX, or FXII, and a polyclonal rabbit antibody (Millipore, #90451) against DNP-hydrazone for carbonyl oxidative modification for human samples. In rat TIC model, we used rabbit polyclonal antibodies against FVII, FX, or FXII, and a goat polyclonal antibody (Bethyl Laboratories, # A150-117A) against DNP-hydrazone. First, the extent of oxidative modification was measured by OxyBlot protein oxidation kit (Millipore, #S7150) following manufacturer's instructions. Protein carbonyl groups were first derivatized to DNP-hydrazone after proteins were immunoprecipitated from trauma patients, rat TIC model or recovered from conditioned media after incubation with activated leukocytes. The DNP-derivatized proteins were then quantified by multiplex fluorescent Western blot analysis. Briefly, these samples were electrophoresed on a 4–12 % gradient SDS-PAGE gel and then transferred to nitrocellulose membranes using a Bio-Rad Mini-Transfer Cell. After incubation with the Intercept blocking buffer (LI-COR, #927–6001) for 1 h at room temperature, membranes were incubated with mouse monoclonal antibodies against albumin (Abcam, 15C7, #AB10241, 1:1000), FVII (Invitrogen, AD-1, #MA5-17635, 1:1000), FX (Enzyme Research, 520, #Mab-FX, 1:1000), FXII (Invitrogen, clone 5A6, #MA5-15902, 1:1000), and rabbit polyclonal antibody against 2,4-dinitrophenylhydrazone (anti-DNP, Millipore, #90451, 1: 150) in case of human samples, or with rabbit antibodies against FVII (Invitrogen, #PA5-115207, 1:1000), FX (Biorbyt, #orb556640, 1:1000), FXII (Proteintech, #12551-1-AP, 1:1000), and Goat polyclonal antibody against 2,4-dinitrophenylhydrazone (anti-DNP, Bethyl Laboratories, # A150-117A, 1: 1000) in case of rat TIC model, respectively, overnight at 4 °C in Intercept antibody diluent (LI-COR, #927–65001). TBS-T (0.2 % Tween 20) was used for all washing steps. After incubation with IRDye 800 CW goat anti-mouse secondary antibody (LI-COR, #926–32210) and IRDye 680 RD goat anti-rabbit secondary antibody (LI-COR, #926–68071) in human samples, or with IRDye 800 CW donkey anti-rabbit IgG secondary antibody (LI-COR, # 926–32213) and IRDye 680 RD donkey anti-goat IgG secondary antibody (LI-COR, # 926–68074) in rat TIC model for 1 h at room temperature, membranes were washed and scanned by using a LI-COR Odyssey Clx. Each band corresponding to FVII, FX and FXII was overlaid with bands detected by anti-DNP to examine for protein-specific oxidation.

### 4.8 In vitro oxidative modification of coagulation factors using leukocytes

To mimic the oxidative stress in blood of trauma patients and to observe its effect on clotting factors, we incubated FVII, FX or FXII with activated human leukocytes. Briefly, fresh whole blood was obtained from healthy human volunteers and red blood cells (RBC) were removed by incubation in ACK RBC lysis buffer (Thermo Fisher Scientific) for 4 min at 37 °C. RBC-lysed one million leukocytes were incubated with 10 ng/mL of IL6 (R&D) to induce activation along with 0.17 μg/mL of FVII (Enzyme Research, #HFV1007), 7 μg/mL of FX (Enzyme Research, #HFX1010) or 31 μg/mL of FXII (Enzyme Research, #HFXII1212) in RPMI 1640 media (Gibco) with or without an antioxidant, vitamin C (100 μmol/L, VetOne, #NDC13985-534-10) or anti-inflammatory melanocortin-derived fusion protein, AQB-565 (100 μmol/L, Aequus BioPharma, synthesized by CSBio, Milpitas, CA)). After 4 h of incubation, conditioned media were collected and subjected to purification of FVII, FX and FXII using immunoprecipitation as mentioned above. Each activity of purified FVII, FX and FXII was measured by the methods as described above. In some cases of measurement of FX activity, we added co-activators, 0.7 IU/mL or 1.4 IU/mL of FVIII (Prospec, #Pro317a) and 3.6 μg/mL of FIX (Enzyme Research, #HFIX1009) to investigate the effects of excess FVIII on the activity of oxidized FX.

### 4.9 Testing effects of individual clotting factor concentration and oxidation on thrombin generation and clot formation by rotational thromboelastometry (ROTEM) using factor deficient plasma

We used clotting factor deficient plasmas (DP) to examine how changes in individual clotting factor concentration and oxidation affect thrombin generation and clot formation. For FV, 6 μg/mL or 10 μg/mL of FV (Abcam, #ab62415) was added into FV DP (Technoclone, #5134004). For FVII, we added 0.17 μg/mL of FVII which was incubated with activated leukocytes or control leukocytes to induce oxidation and purified by immunoprecipitation as described above in FVII DP (Technoclone, #5144015). In the case of FVIII, 0.7 IU/ml or 1.4 IU/mL of FVIII were added in FVIII DP (Technoclone, #5154007). In the case of FX, we added 7 μg/mL of FX after oxidation by incubation with activated leukocytes or control leukocytes and purified by immunoprecipitation to FX DP (Technoclone, #5174004). For FXII, 31 μg/mL of FXII which was oxidized by incubation with activated leukocytes or control leukocytes and purified by immunoprecipitation and was added in FXII DP (Technoclone, #5194008). To investigate the ability of FVIII to compensate for factor oxidation, additional 0.7 UI/mL of FVIII were added with FV, FVII, FX or FXII in FV DP, FVII DP, FX DP and FXII DP. To mimick the pattern of clotting factor oxidation noted in trauma patients, we used 6 μg/mL of FV, 0.17 mg/mL of oxidized FVII, 1.4 IU/mL of FVIII, 3.6 μg/mL of FIX, 7 μg/mL of oxidized FX, 2.2 μg/mL of FXI (Enzyme Research, # HFXI1111), 31 μg/mL of oxidized FXII and 10 μg/mL of prothrombin (Enzyme Research, #HP1002) in HEPES buffer (44 mmol/L HEPES and 140 mmol/L NaCl, pH 7.4), and control reaction including including 10 μg/mL of FV, 0.17 mg/mL of control unoxidized FVII, 0.7 IU/mL of FVIII, 3.6 μg/mL of FIX, 7 μg/mL of control unoxidized FX, 2.2 μg/mL of FXI (Enzyme Research, # HFXI1111), 31 μg/mL of control unoxidized FXII and 10 μg/mL of prothrombin (Enzyme Research, #HP1002) in HEPES buffer. TGA and ROTEM (Werfern) using EXTEM (tissue factor triggered extrinsic pathway, FV, FVII, FVIII and FX) or INTEM (ellagic acid triggered intrinsic pathway, FXII) assays were used to measure thrombin generation and clot formation, respectively. ROTEM assays (Werfen) were performed over 60 min and parameters included clotting time (CT), defined as the time between reagent addition to clot formation, α-angle, which reflects the rate of clot formation, maximum clot firmness (MCF), lysis index-30 min (LI-30), the percentage of MCF retained 30 min after initiation of clot formation, and maximum lysis (ML), the percentage of clot strength lost compared to the MCF at the end of analysis.

### 4.10 In vivo study using rat polytrauma coagulopathy model

We used an established rat model of trauma and hemorrhagic shock to investigate the importance of clotting factor oxidation after trauma and the potential for anti-inflammatory treatment using an anti-inflammatory-derived fusion protein (AQB-565) that demonstrated anti-oxidant and anti-inflammatory effects on fibrinogen in previous work [17]. Briefly, male Sprague-Dawley rats (Charles River) between 270 and 450 g were traumatized and blood was drawn to achieve hemorrhagic shock as described previously [21]. Fifteen minutes after hemorrhage, animals were dosed with either AQB-565 (1.0 mg/kg reconstituted in 0.9 % saline, total volume ∼1.5–2.0 mL), or volume-matched saline control (Dechra Veterinary). Infusions were continuously administered over 15 min via syringe pump (KD Scientific, Huizen, Netherlands). Animals were observed with no intervention until 60 min past the initiation of hemorrhage, at which time they were reinfused with the autologous citrated whole blood removed during hemorrhage. Blood was infused at a rate of 1 mL/kg/minute to maintain goal mean arterial pressure (MAP) = 60 mmHg. Rats were observed and then euthanized at 180 min post-hemorrhage by exsanguination under isoflurane. Blood samples were taken at baseline prior to injury and at 180 min of trauma/shock for analysis. The plasma concentration of AQB-565 was measured by human ACTH ELISA Kit (Abcam, #ab267814). For this, we used AQB-565 to make the standard curve for calculating plasma AQB-565 concentrations.

### 4.11 Sex as a biological variable

We used male Sprague-Dawley rats in polytrauma coagulopathy model since female rats have been shown hypercoagulability and resistant to coagulopathy [58]. We enrolled both male and female patients. However, trauma predominantly affects males (>70 %), so the clinical trauma studies typically enroll more males than females which is an accurate reflection of those most affected in the U.S. population.

### 4.12 Statistics

Statistical tests were selected based upon data distribution as appropriate using SPSS (Windows version 19) or OriginPro software (version 8.6; Origin Laboratory). All data are shown as means ± SEM. Normality and Homogeneity of variance were tested using the Shapiro-Wilk and Brown-Forsythe tests. Student's t-test was used to detect differences within groups (2-tailed) if the data were normally distributed with homogeneity of variance; otherwise, when normality was not met, the Mann-Whitney *U* test was applied. One-way ANOVA (ANOVA) was used to compare differences between groups with Tukey multiple comparison post-hoc adjustment for multiple comparisons if the data were normally distributed with homogeneity of variance. Otherwise, Kruskal-Wallis with Dunn multiple comparison test was used when data were not normally distributed. Pearson coefficient was used for linear regression analysis. An overall p value < 0.05 was considered statistically significant.

### 4.13 Study approval

All patients and donors provided written informed consent and authorization for blood draws and release of medical information according to institutional human subjects’ board approved protocols (Protocol # BT001 and STUDY00008295). All *in vivo* rat experimental procedures were reviewed and approved by the Institutional Animal Care and Use Committee of the University of Washington (Protocol #4329-05) and the U.S. Department of Defense Animal Care and Use Review Office.

## CRediT authorship contribution statement

**Chang Yeop Han:** Data curation, Formal analysis, Investigation, Methodology, Visualization, Writing – original draft, Writing – review & editing. **Alexander E. St John:** Data curation, Funding acquisition, Investigation, Writing – review & editing. **Jung Heon Kim:** Data curation, Writing – review & editing. **Xu Wang:** Data curation, Investigation, Methodology, Writing – review & editing. **Kristyn M. Ringgold:** Data curation, Investigation, Methodology, Writing – review & editing. **Lauren E. Neidig:** Data curation, Investigation, Methodology, Writing – review & editing. **Ronald Berenson:** Funding acquisition, Resources, Writing – review & editing. **Susan A. Stern:** Funding acquisition, Resources, Supervision, Writing – review & editing. **Nathan J. White:** Conceptualization, Data curation, Formal analysis, Funding acquisition, Project administration, Resources, Supervision, Writing – original draft, Writing – review & editing.

## Declaration of competing interest

N.J.W. has received funding from U.S. NIH, DoD, and is shareholder in Stasys Medical Corp, and consultant to Velico Medical Corp. and Grifols Inc. All other authors declare no conflict of interest.

## Data Availability

Data will be made available on request.
