# Supplementary material for: Inflammation contributes to trauma-induced coagulopathy by oxidation of multiple clotting factors
Source: Redox Biol. 2025 Nov 30;89:103956. doi: 10.1016/j.redox.2025.103956 (PMC12721292; doi:10.1016/j.redox.2025.103956)
Supplement: Multimedia component 1 [file mmc1.pdf]

Supplemental Materials

Supplemental Table 1. Demographic Features of Study Subjects with trauma and healthy controls

Characteristics	Patients (n=21) Mean ± SD	Controls (n=10) Mean ± SD	P value
Female	2	2	0.20
Male	19	8	
Age (yrs)	44.9 ± 18.9	47.1 ± 18.5	0.69
ISS	19.1 ± 12.8	n/a	
RTS	5.3 ± 1.8	n/a	
Mechanism, blunt (%)	11 (52%)	n/a	
Gunshot wound, n (%)	8 (38%)	n/a	
Fall, n (%)	4 (19%)	n/a	
Motor vehicle collision, n (%)	2 (10%)	n/a	
Motorcycle collision, n (%)	2 (10%)	n/a	
Pedestrian struck, n (%)	2 (10%)	n/a	
Stab wound, n (%)	2 (10%)	n/a	
Bicycle collision, n (%)	1 (5%)	n/a	
Initial vital signs			
Heart rate (beats/min)	94 ± 16	n/a	
Systolic blood pressure (mmHg)	130 ± 21	n/a	
Diastolic blood pressure (mmHg)	92 ± 19	n/a	
SI	0.7 ± 0.2	n/a	
Initial laboratory values			
Hemoglobin (mg/dL)	13.4 ± 1.7	n/a	

Hematocrit (%)	41 ± 5	n/a
Platelets (x10^9/L)	259 ± 55	n/a
INR	1.16 ± 0.25	n/a
Lactate (mmol/L)	2.4 ± 1.8	n/a
24-hour transfusion requirement (units)	1.0 ± 1.9	n/a
Mortality, n (%)	3 (14%)	n/a

n/a = not applicable

Supplemental Figures

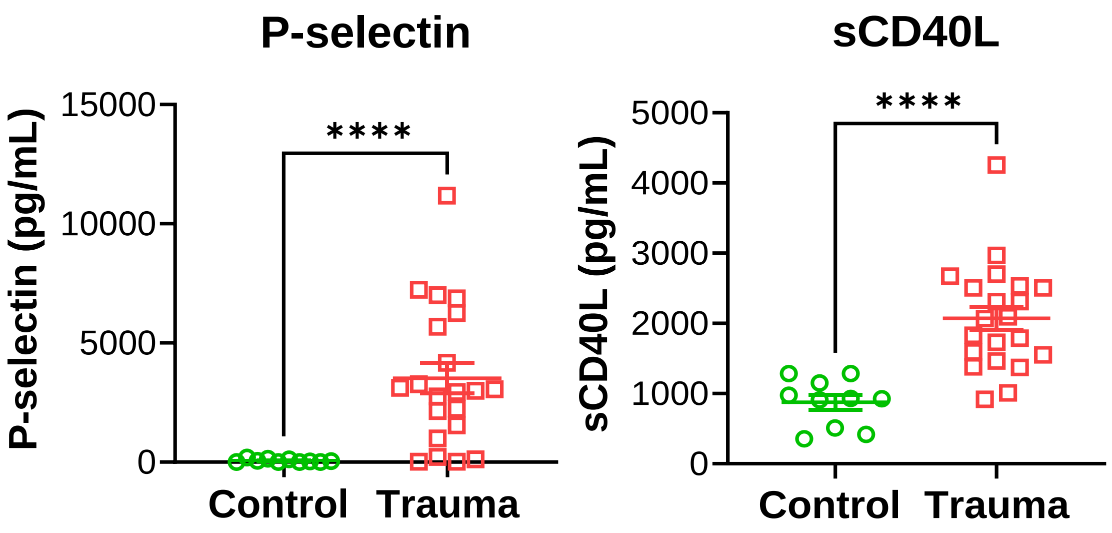

Supplemental Figure 1. Trauma induces the damage in endothelial cells. Human plasma was collected from trauma patients (n=21) and healthy controls (n=10). P-selectin and sCD40L were measured as described in Methods (mean ± standard error of the mean (SEM)). ****p< 0.0001 vs. control. Mann-Whitney U test.

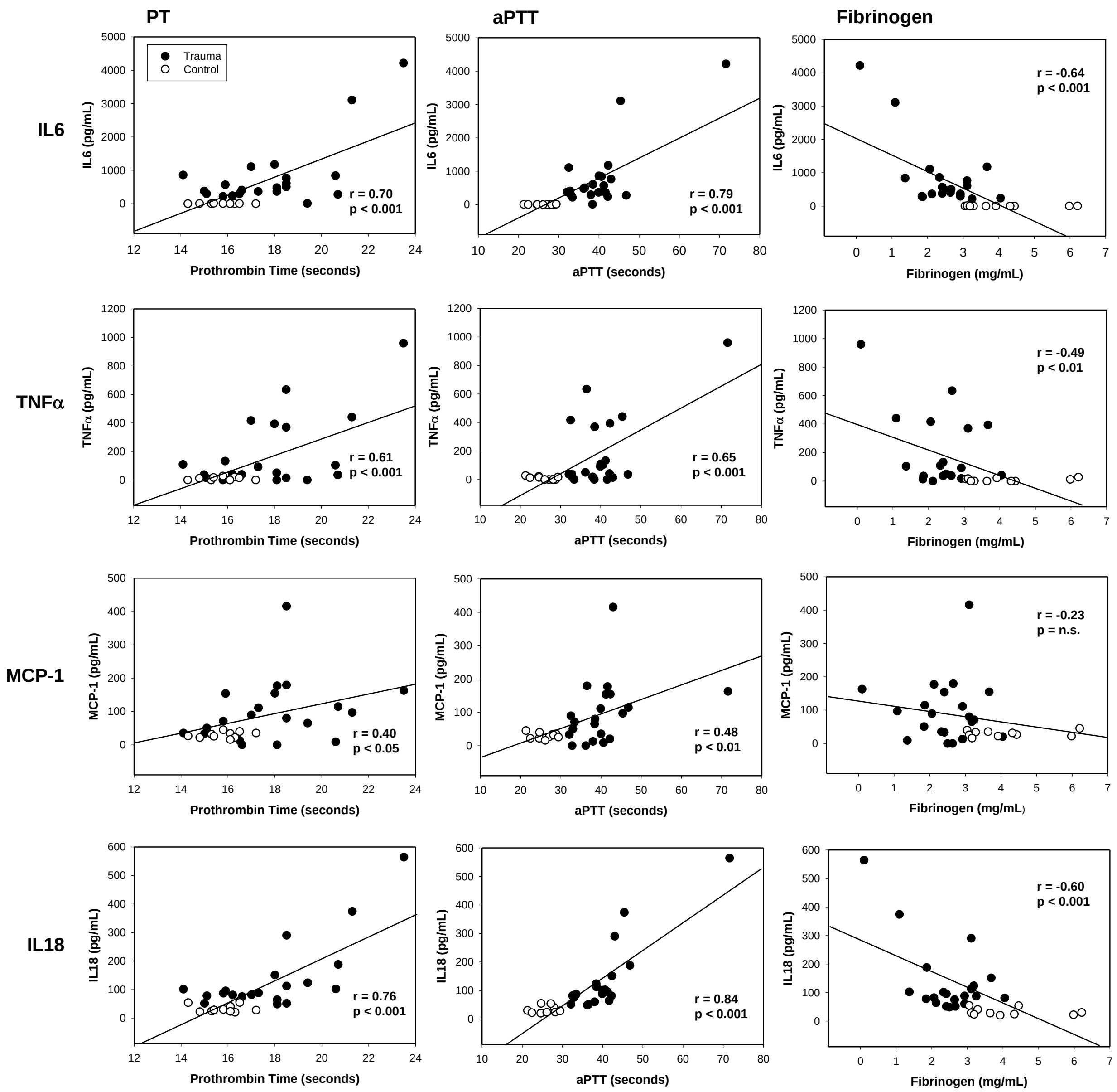

Supplemental Figure 2. Trauma-induced coagulopathy relates with inflammation in humans. Linear regression relationships among IL6, TNFα, MCP-1, IL18 and PT, aPTT or fibrinogen from trauma patients (n=21, closed circle) and healthy controls (n=10, open circle) were plotted using Pearson correlation coefficient.

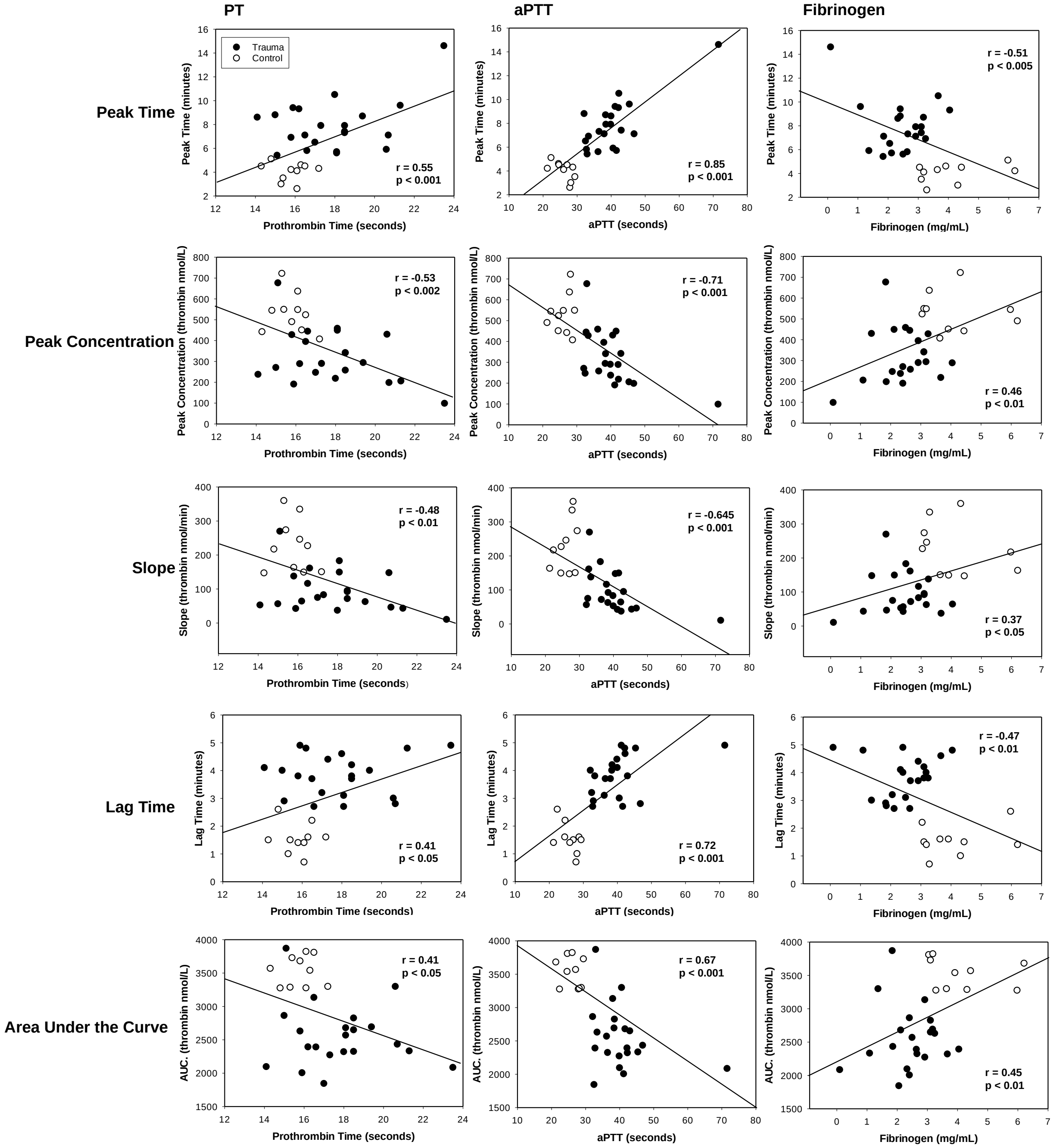

Supplemental Figure 3. TIC correlates with thrombin generation in humans. Linear regression relationships among peak time, peak concentration, slope, lag time, AUC. in TGA and PT, aPTT or fibrinogen from trauma patients (n=21, closed circle) and healthy controls (n=10, open circle) were plotted using Pearson correlation coefficient.

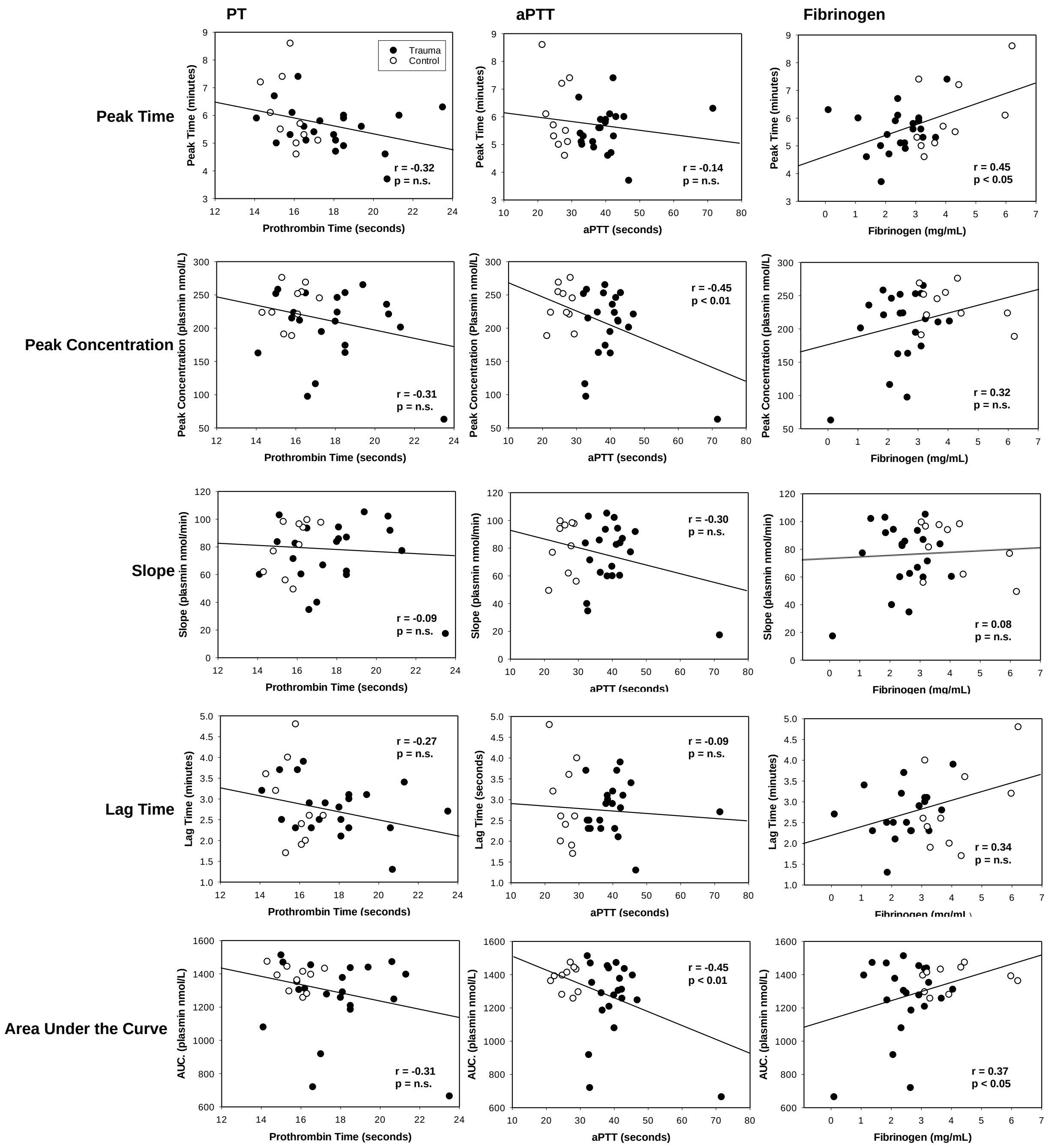

Supplemental Figure 4. TIC has very weak or no relationship with plasmin generation in humans. Linear regression relationships among peak time, peak concentration, slope, lag time, AUC. in PGA and PT, aPTT or fibrinogen from trauma patients (n=21, closed circle) and healthy controls (n=10, open circle) were plotted using Pearson correlation coefficient.

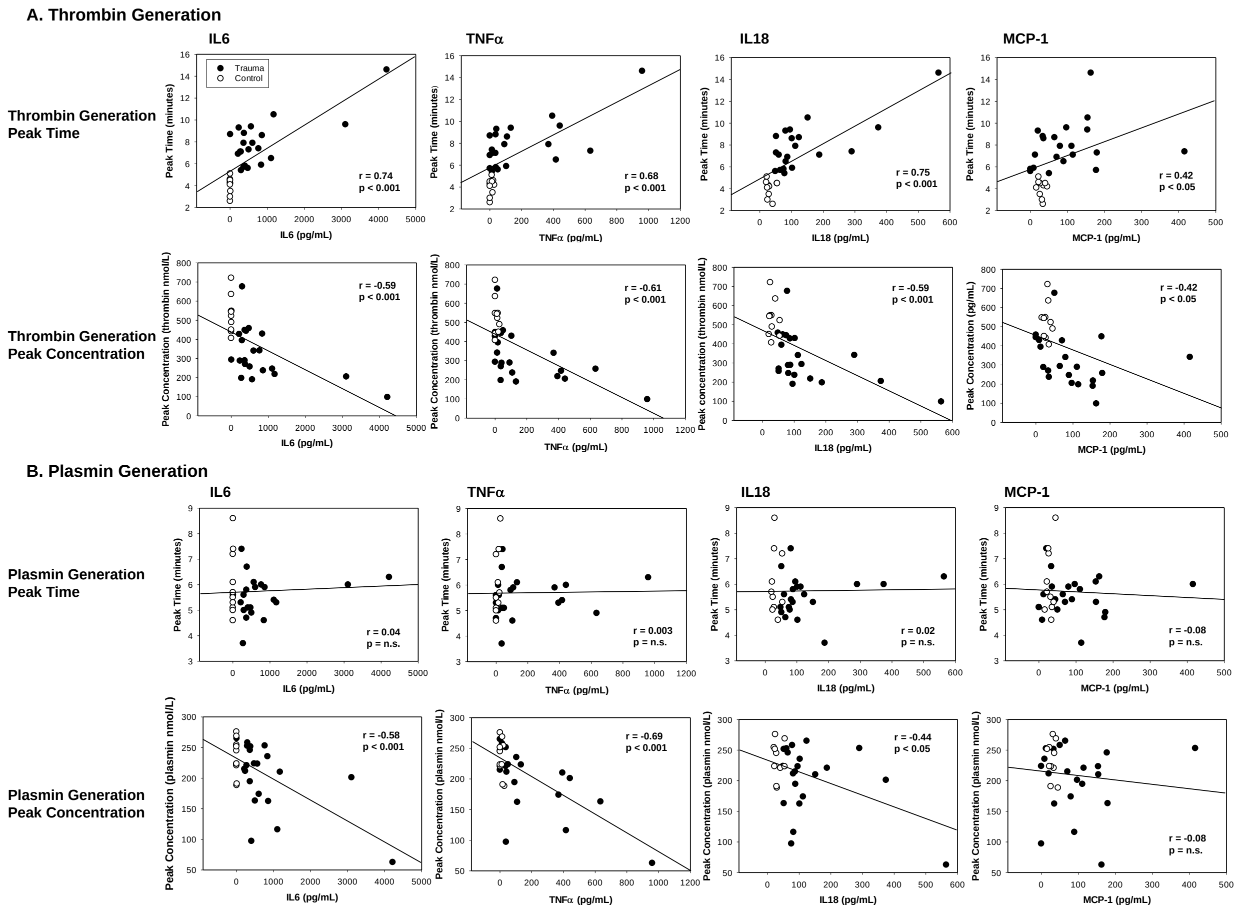

Supplemental Figure 5. Thrombin generation highly correlates with proinflammatory cytokines in trauma patients, while plasmin generation does not. Linear regression relationships among peak time, peak concentration in TGA (**A**) or PGA (**B**) and IL6, TNFα, IL18, or MCP-1 from trauma patients (n=21, closed circle) and healthy controls (n=10, open circle) were plotted using Pearson correlation coefficient.

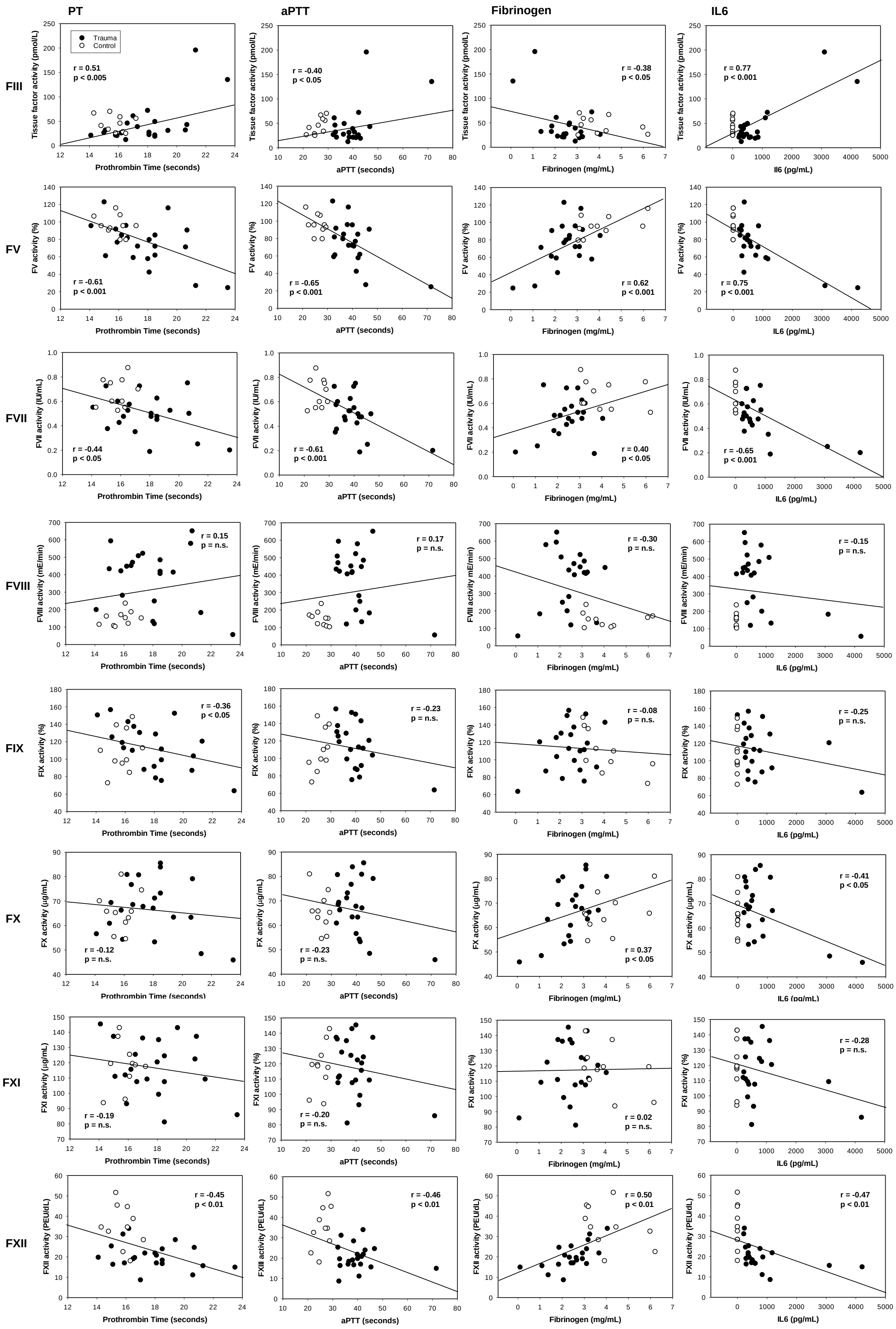
PT
aPTT
Fibrinogen
IL6
FIII
FV
FVII
FVIII
FIX
FX
FXI
FXII
Trauma
Control
Tissue factor activity (pmol/L)
FV activity (%)
FVII activity (IU/mL)
FVIII activity (mE/min)
FIX activity (%)
FX activity (µg/mL)
FXI activity (µg/mL)
FXI activity (%)
FXII activity (PEU/dL)
Prothrombin Time (seconds)
aPTT (seconds)
Fibrinogen (mg/mL)
Il6 (pg/mL)
IL6 (pg/mL)
r = 0.51
p < 0.005
r = -0.40
p < 0.05
r = -0.38
p < 0.05
r = 0.77
p < 0.001
r = -0.61
p < 0.001
r = -0.65
p < 0.001
r = 0.62
p < 0.001
r = 0.75
p < 0.001
r = -0.44
p < 0.05
r = -0.61
p < 0.001
r = 0.40
p < 0.05
r = -0.65
p < 0.001
r = 0.15
p = n.s.
r = 0.17
p = n.s.
r = -0.30
p = n.s.
r = -0.15
p = n.s.
r = -0.36
p < 0.05
r = -0.23
p = n.s.
r = -0.08
p = n.s.
r = -0.25
p = n.s.
r = -0.12
p = n.s.
r = -0.23
p = n.s.
r = 0.37
p < 0.05
r = -0.41
p < 0.05
r = -0.19
p = n.s.
r = -0.20
p = n.s.
r = 0.02
p = n.s.
r = -0.28
p = n.s.
r = -0.45
p < 0.01
r = -0.46
p < 0.01
r = 0.50
p < 0.01
r = -0.47
p < 0.01

Supplemental Figure 6. FIII, FV, FVII and FXII correlate with coagulopathy and inflammation in trauma patients, while FVIII, FIX and FX do not, Linear regression relationships among the activities of FIII, FV, FVII, FVIII, FIX, FX, FXI and FXII and PT, aPTT, fibrinogen or IL6 from trauma patients (n=21, closed circle) and healthy controls (n=10, open circle) were plotted using Pearson correlation coefficient.

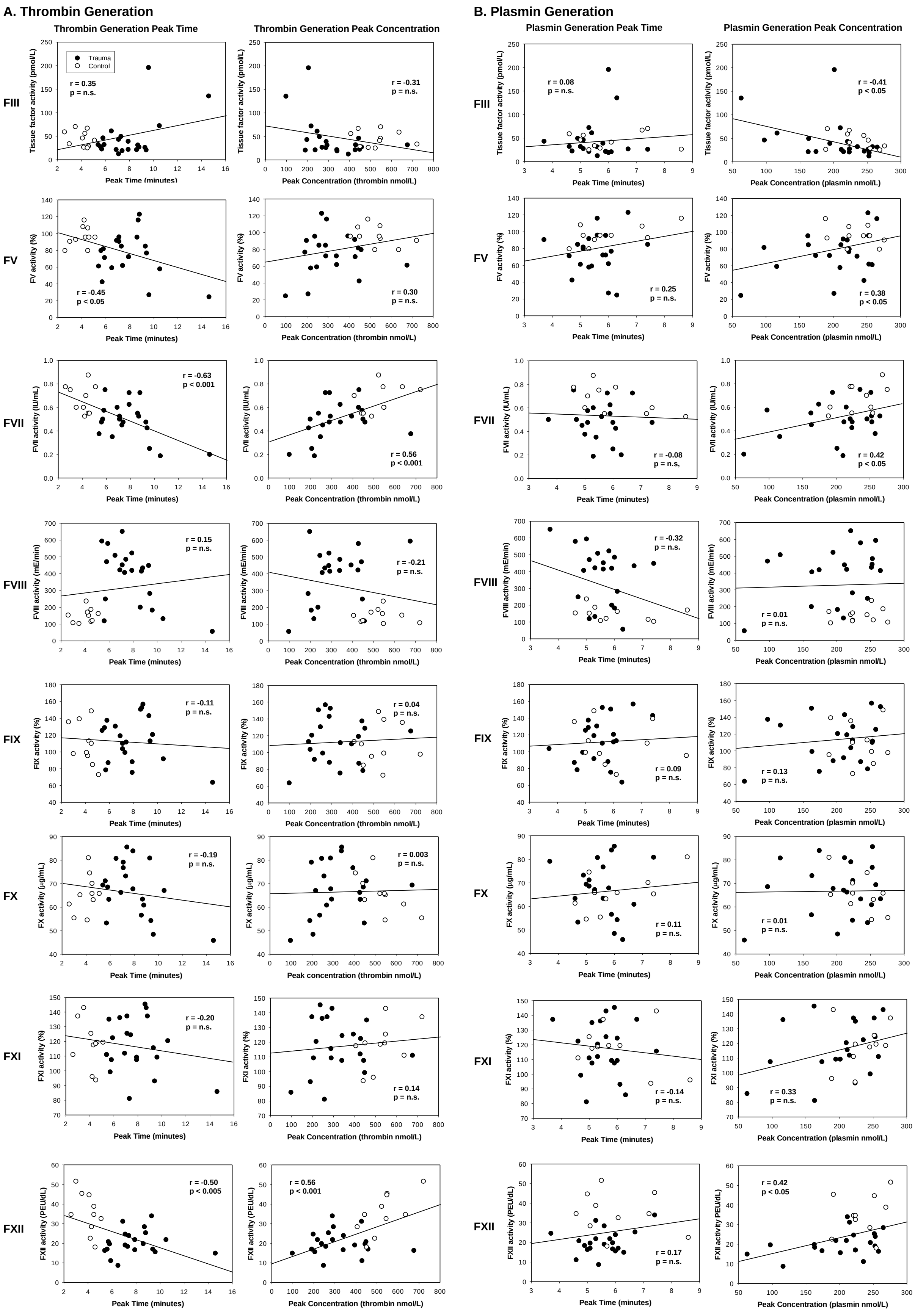
A. Thrombin Generation
B. Plasmin Generation
Thrombin Generation Peak Time
Thrombin Generation Peak Concentration
Plasmin Generation Peak Time
Plasmin Generation Peak Concentration
Trauma
Control
FIII
r = 0.35
p = n.s.
r = -0.31
p = n.s.
r = 0.08
p = n.s.
r = -0.41
p < 0.05
FV
r = -0.45
p < 0.05
r = 0.30
p = n.s.
r = 0.25
p = n.s.
r = 0.38
p < 0.05
FVII
r = -0.63
p < 0.001
r = 0.56
p < 0.001
r = -0.08
p = n.s,
r = 0.42
p < 0.05
FVIII
r = 0.15
p = n.s.
r = -0.21
p = n.s.
r = -0.32
p = n.s.
r = 0.01
p = n.s.
FIX
r = -0.11
p = n.s.
r = 0.04
p = n.s.
r = 0.09
p = n.s.
r = 0.13
p = n.s.
FX
r = -0.19
p = n.s.
r = 0.003
p = n.s.
r = 0.11
p = n.s.
r = 0.01
p = n.s.
FXI
r = -0.20
p = n.s.
r = 0.14
p = n.s.
r = -0.14
p = n.s.
r = 0.33
p = n.s.
FXII
r = -0.50
p < 0.005
r = 0.56
p < 0.001
r = 0.17
p = n.s.
r = 0.42
p < 0.05
Tissue factor activity (pmol/L)
FV activity (%)
FVII activity (IU/mL)
FVIII activity (mE/min)
FIX activity (%)
FX activity (µg/mL)
FXI activity (%)
FXII activity (PEU/dL)
Peak Time (minutes)
Peak Concentration (thrombin nmol/L)
Peak Concentration (plasmin nmol/L)

Supplemental Figure 7. FV, FVII and FXII correlate with thrombin generation, not plasmin generation in trauma patients, while FIII, FVIII, FIX and FX do not, Linear regression relationships among the activities of FIII, FV, FVII, FVIII, FIX, FX, FXI and FXII and peak time or peak concentration in TGA (**A**), or peak time or peak concentration in PGA (**B**) from trauma patients (n=21, closed circle) and healthy controls (n=10, open circle) were plotted using Pearson correlation coefficient.

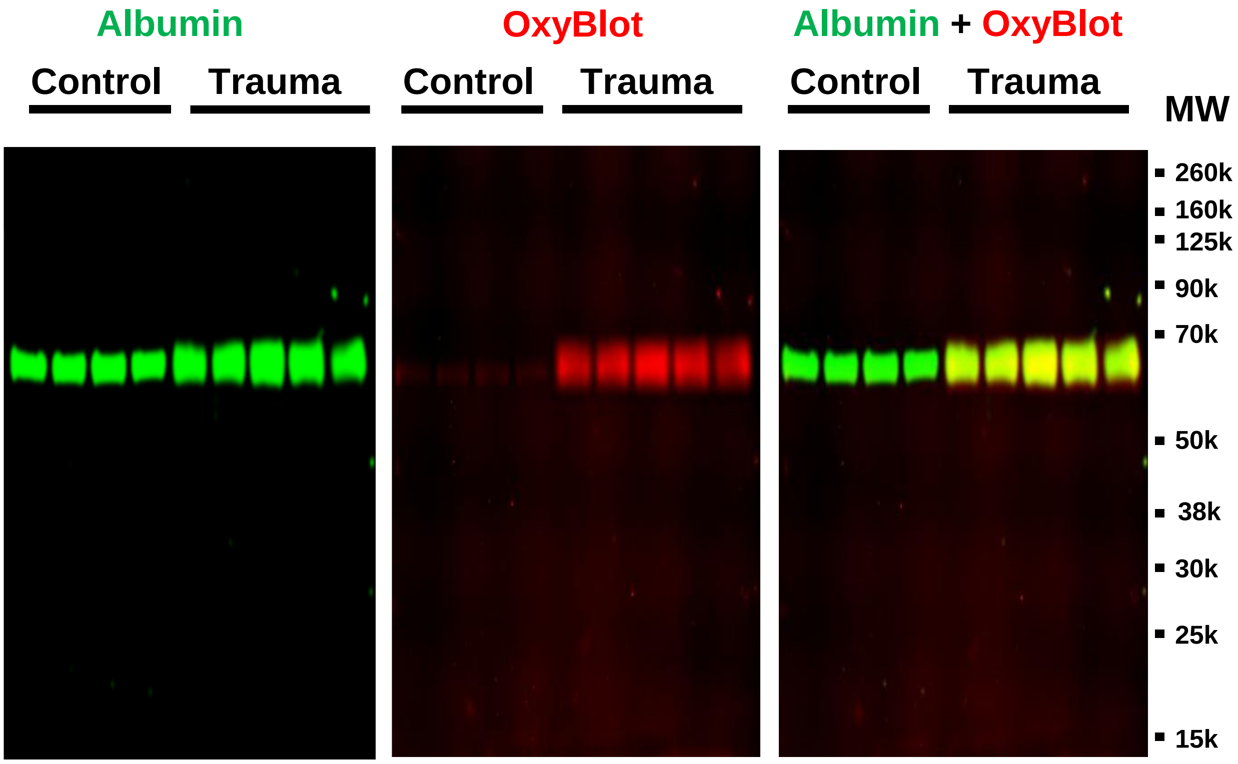

Supplemental Figure 8. Albumin is oxidized in trauma patients. Albumin was immunoprecipitated using monoclonal antibodies against albumin from plasma of trauma patient (n=5) and healthy controls (n=4) as described in Methods. Purified albumin was analyzed by OxyBlot and immunoblotting using albumin – specific antibodies. Representative blots are displayed.

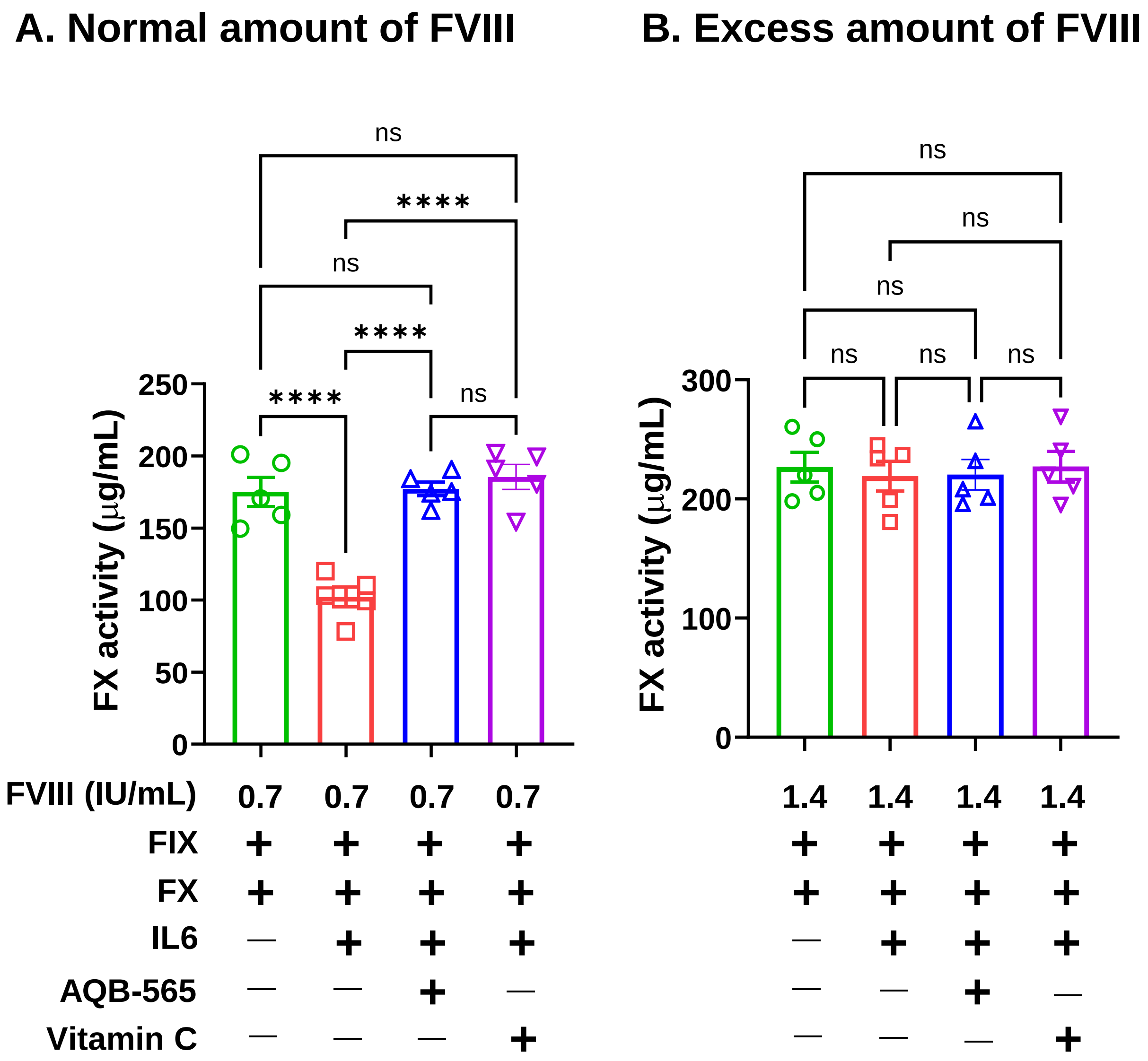

Supplemental Figure 9. Excess FVIII reverses the effect of oxidation, whereas the activity of FX is reduced. FX (n=5) purified from conditioned media as described in Figure 6 and Methods was incubated with 0.7 IU/ml of FVIII (normal concentration, **A**), or 1.4 IU/ml of FVIII (excess concentration, **B**) and 3.6 μg/mL of FIX. After incubation, the activity of FX was measured. Data represent mean ± SEM. ns= not significant. ****p < 0.0001 vs. each group. ANOVA and Tukey post-hoc test.

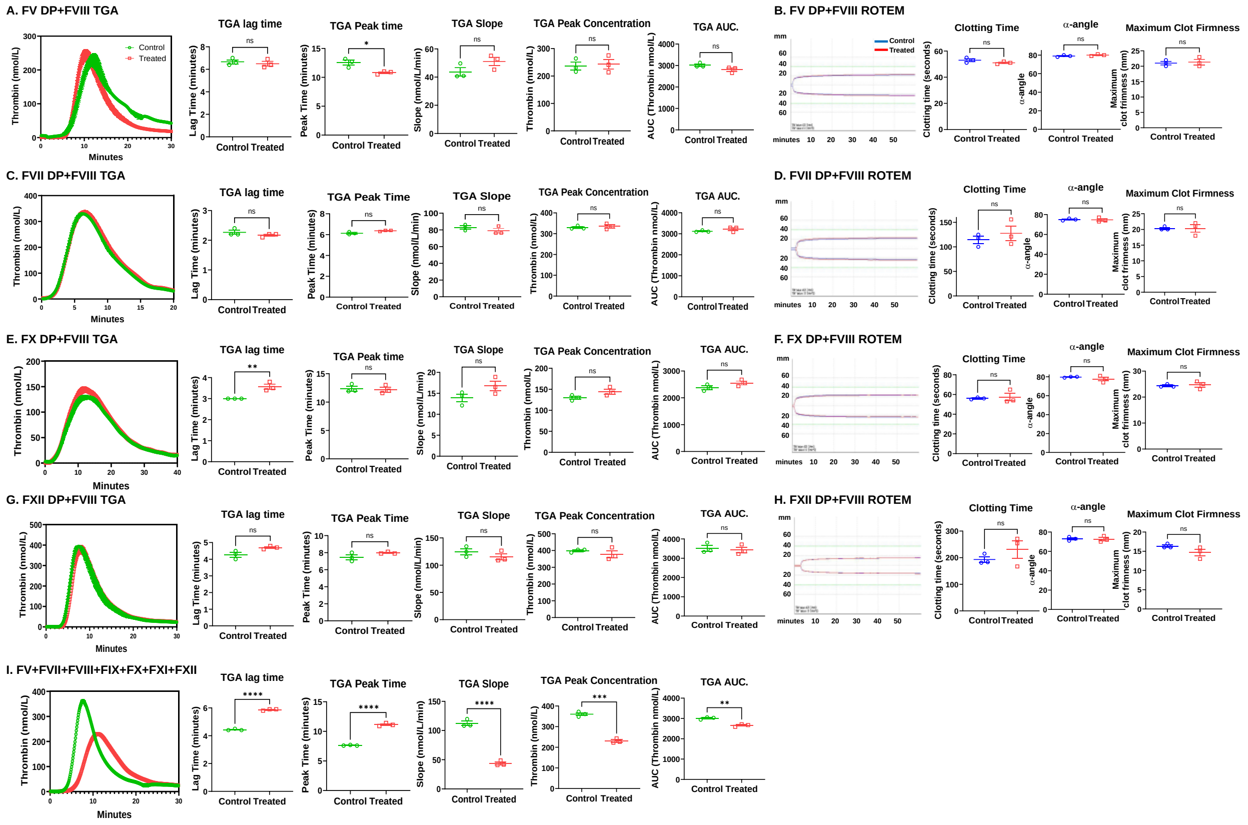

Supplemental Figure 10. Excess FVIII compensates for individual clotting factor dysfunction but does not recover multiple clotting factor dysfunction noted in trauma patients. TGA (**A**, **C**, **E**, and **G**) and ROTEM (**B**, **D**, **F**, and **H**) were performed using each coagulation factor-specific DP (n=3) as described in Methods and Figure 7. **A** and **B**. control amount (control) of FV which we found in healthy controls or reduced amount (treated) of FV supplemented with excess of FVIII were added in FV DP. **C** and **D**. control FVII (control) and oxidized FVII (treated) supplemented with excess of FVIII were added to FVII DP. **E** and **F**. control FX (control) and oxidized FX (treated) supplemented with excess of FVIII were added to FX DP. **G** and **H**. control FXII (control) and oxidized FXII (treated) supplemented with excess of FVIIIs were added to FXII DP. **I**. an *in vitro* mixture (n=3) of reduced amount of FV, oxidized FVII, excess amount of FVIII, control amount of FIX, oxidized FX and oxidized FXII was

prepared as described in Methods and analyzed with TGA. Data represent mean ± SEM. *p< 0.05, **p< 0.01, ***p<0.001, ****p < 0.0001 vs. control. Student's t-test.

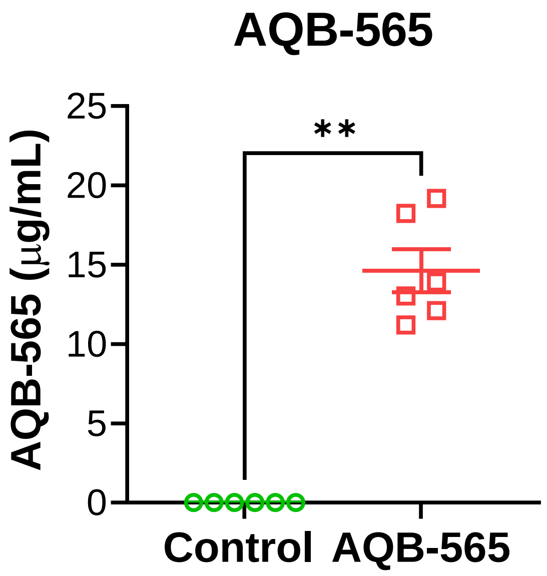

Supplemental Figure 11. AQB-565 concentration in rat plasma samples. Polytrauma-induced rats were injected with saline (control, n=6) or AQB-565 (treatment, n=6) as described in Methods. The concentration of AQB-565 was determined using human ACTH ELISA. Data represent mean ± SEM. $^{**}p < 0.01$ vs. control. Mann-Whitney U test.
