# Supplementary material for: Inflammation contributes to trauma-induced coagulopathy by oxidation of multiple clotting factors
Source: Redox Biol. 2025 Nov 30;89:103956. doi: 10.1016/j.redox.2025.103956 (PMC12721292; doi:10.1016/j.redox.2025.103956)
Supplement: Multimedia component 3 [file mmc3.pdf]

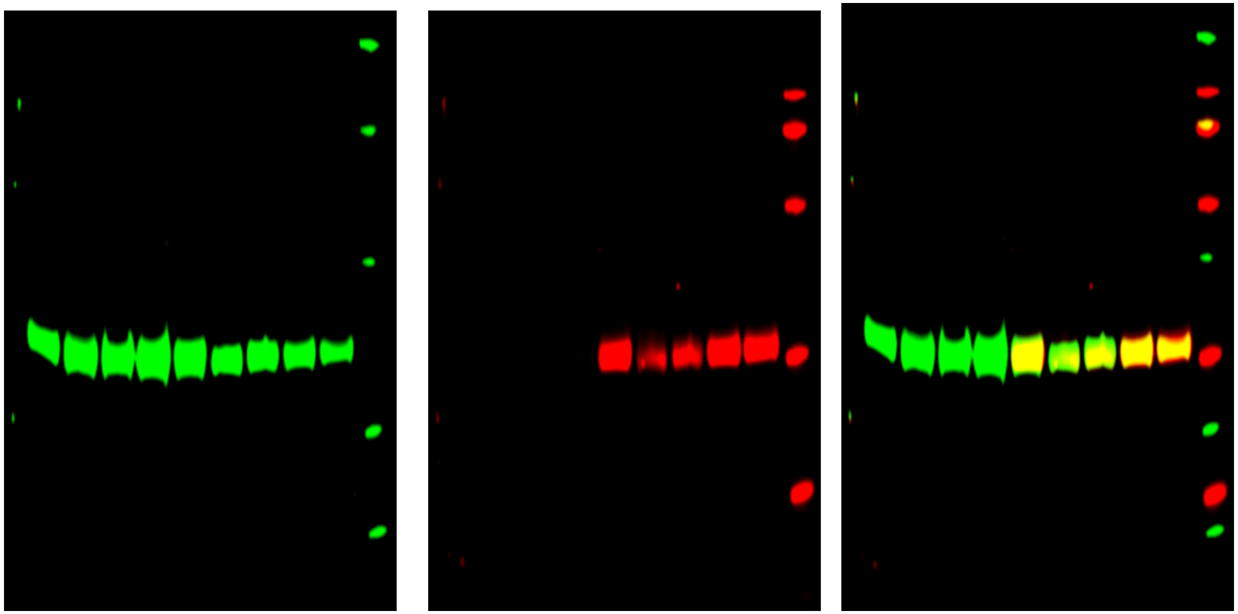

Figure 5A

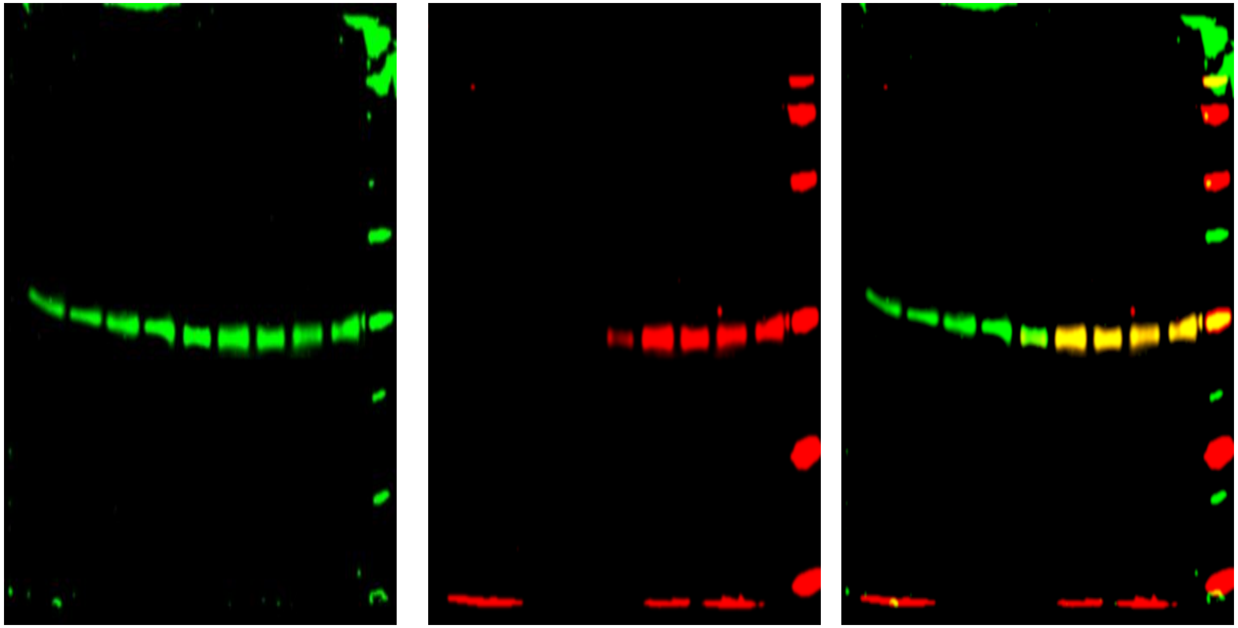

Figure 5B

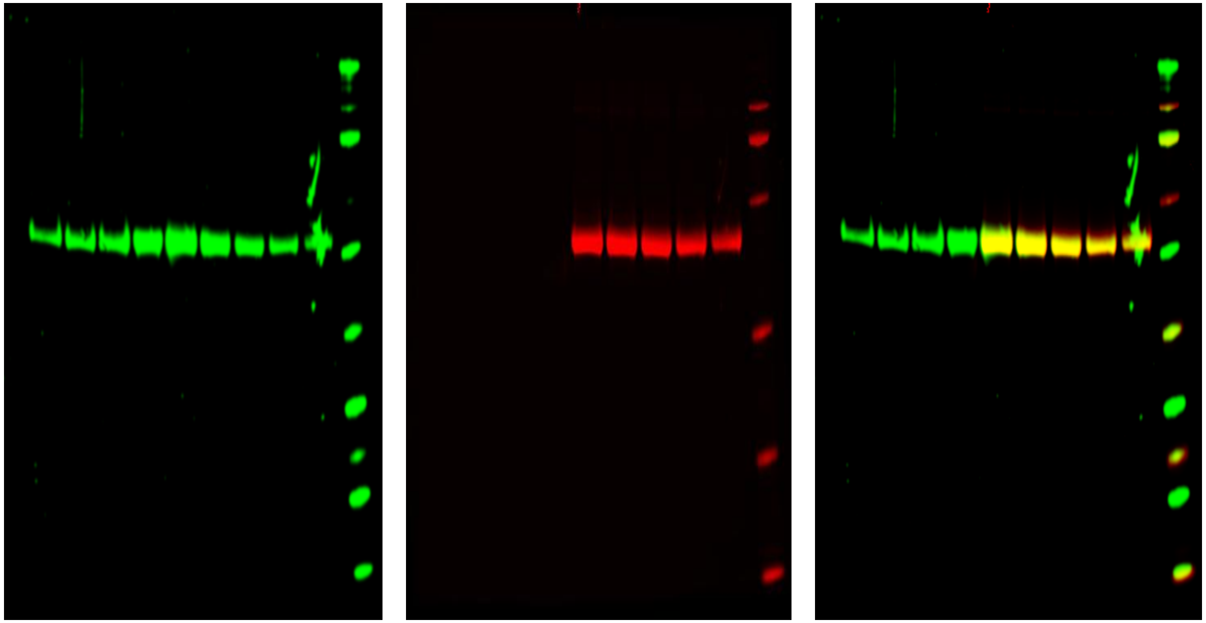

Figure 5C

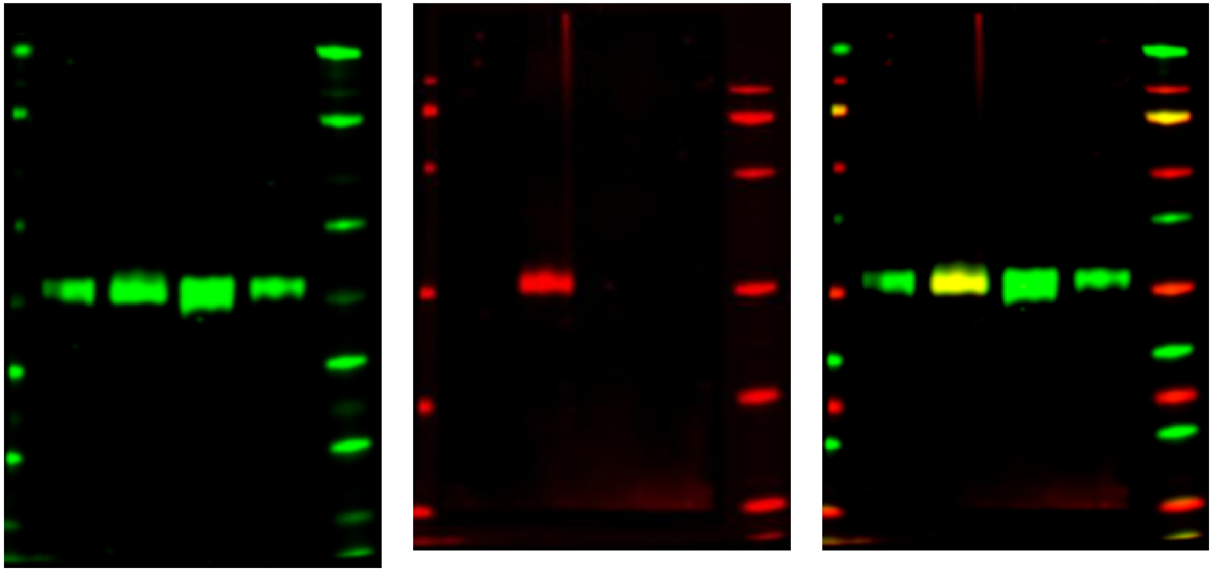

Figure 6A

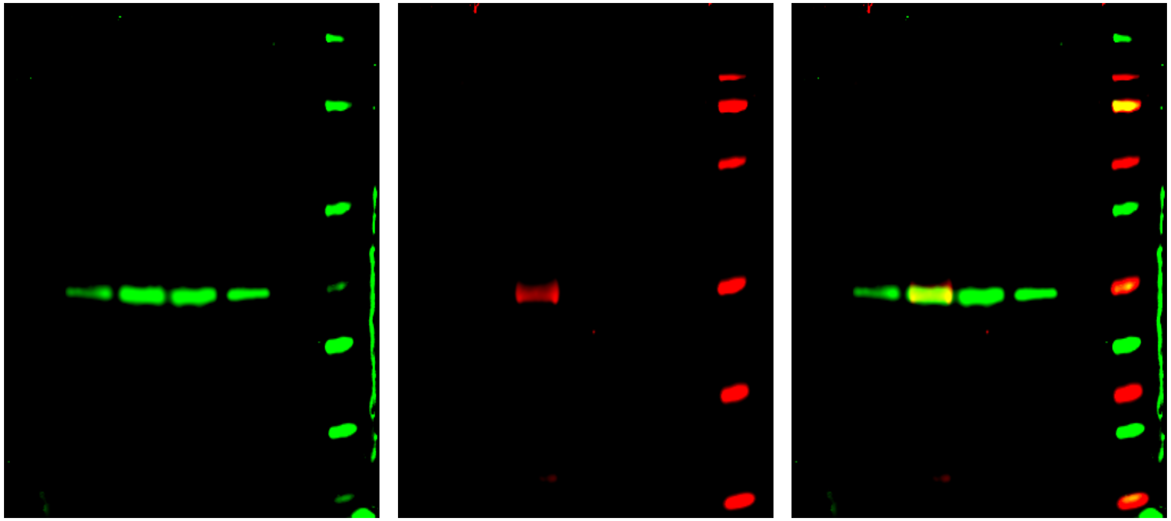

Figure 6B

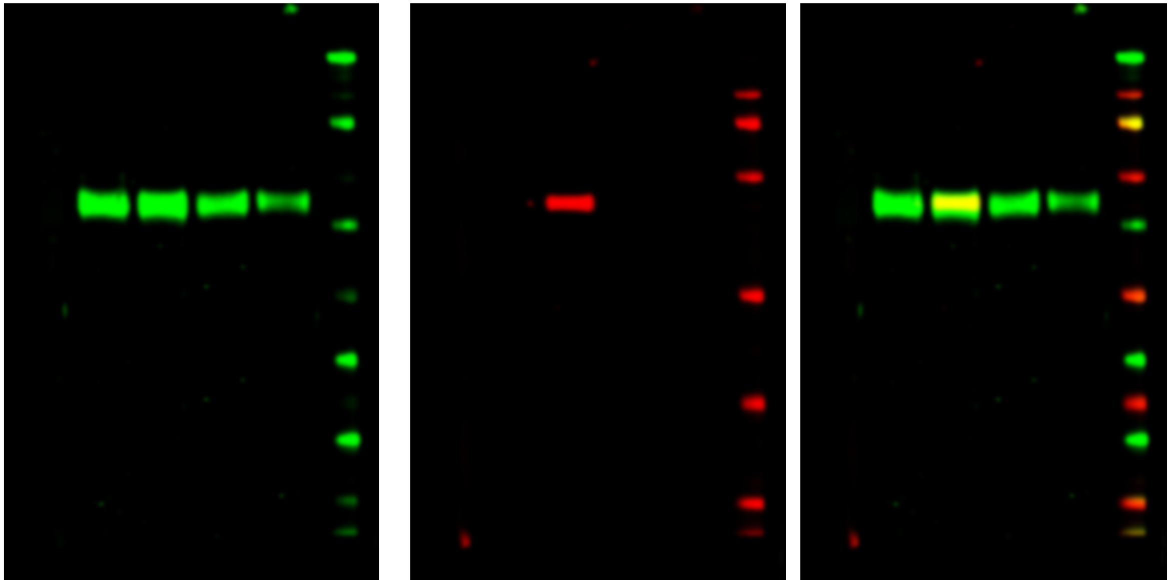

Figure 6C

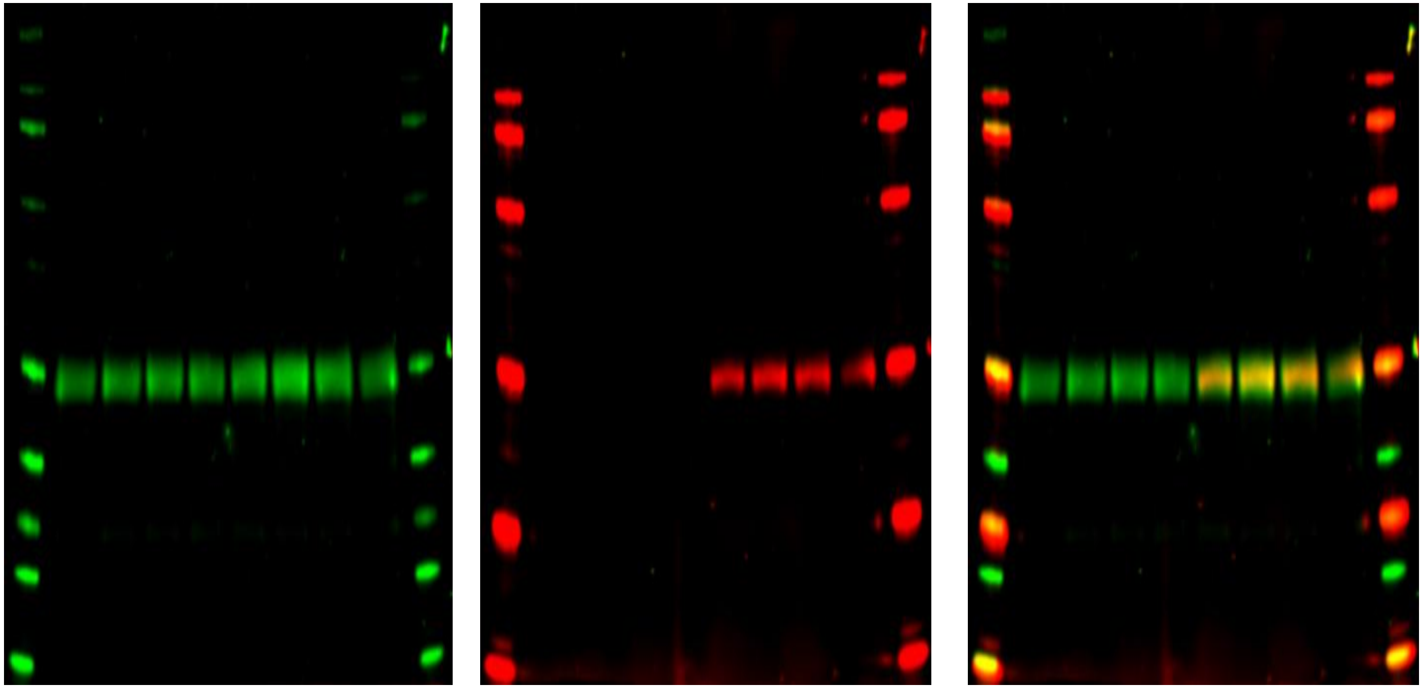

Figure 8D

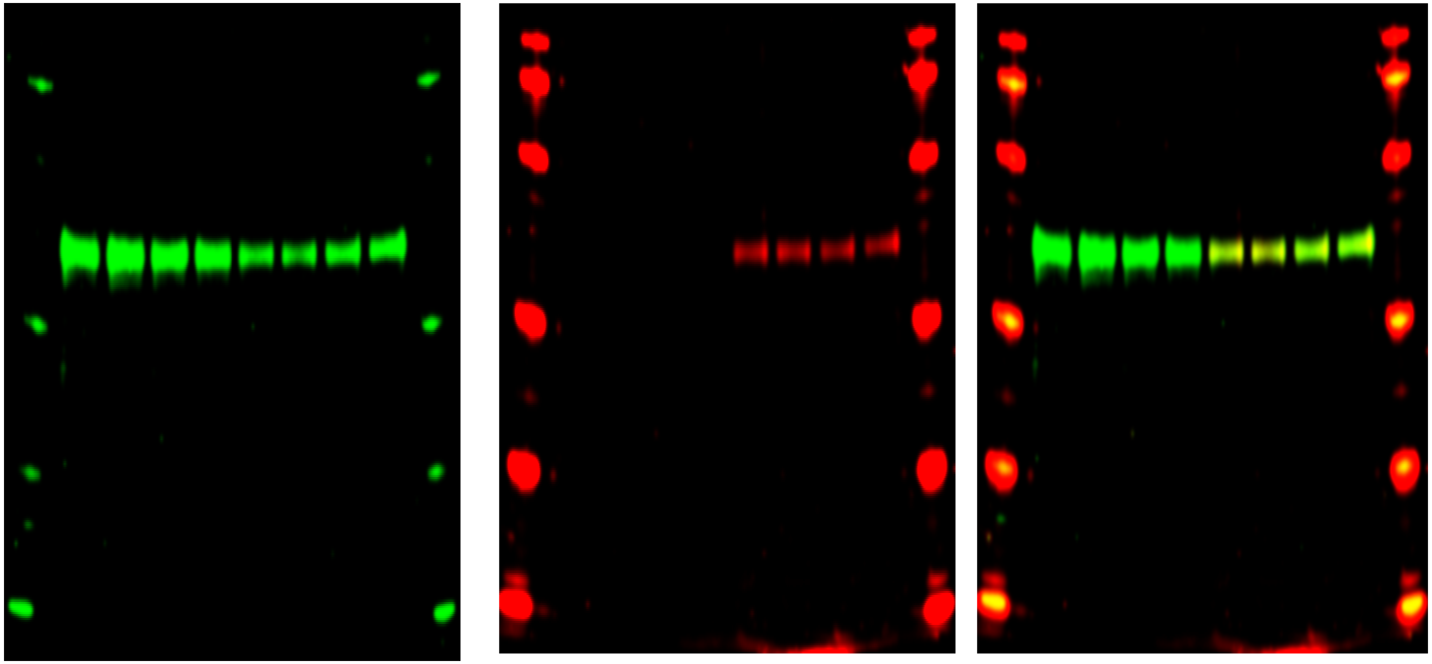

Figure 8E

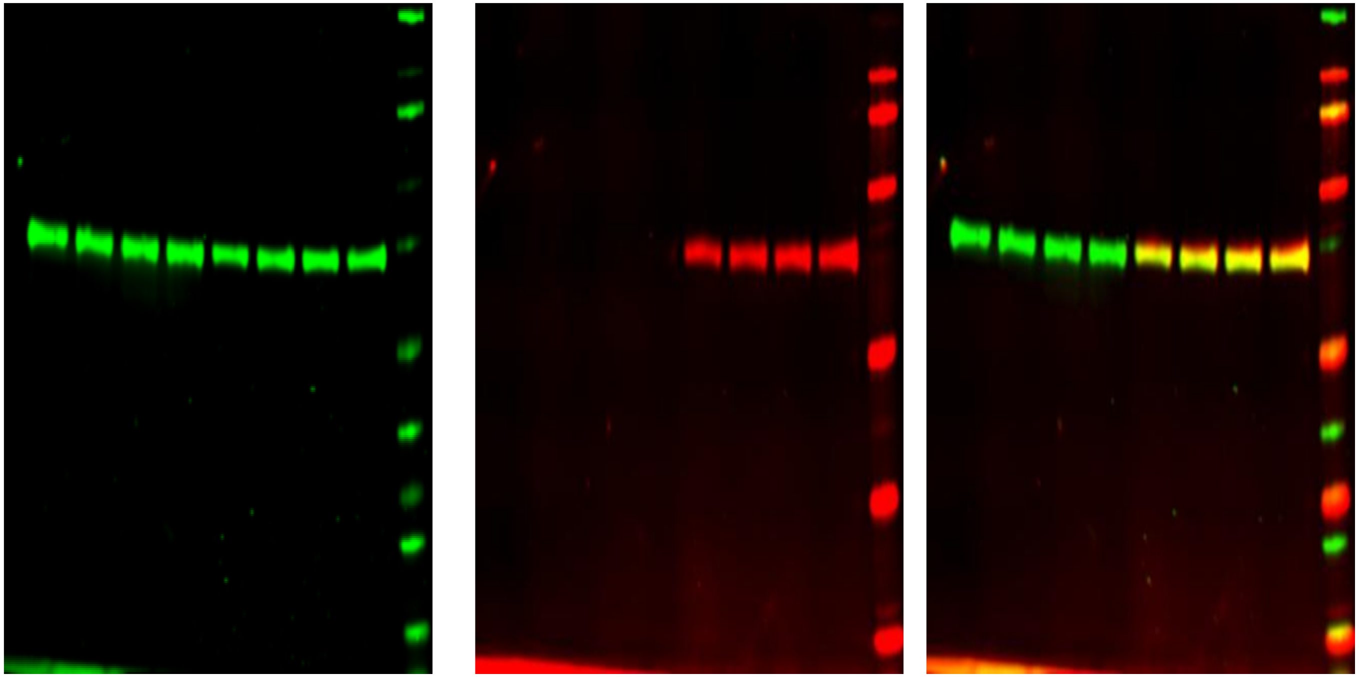

Figure 8F

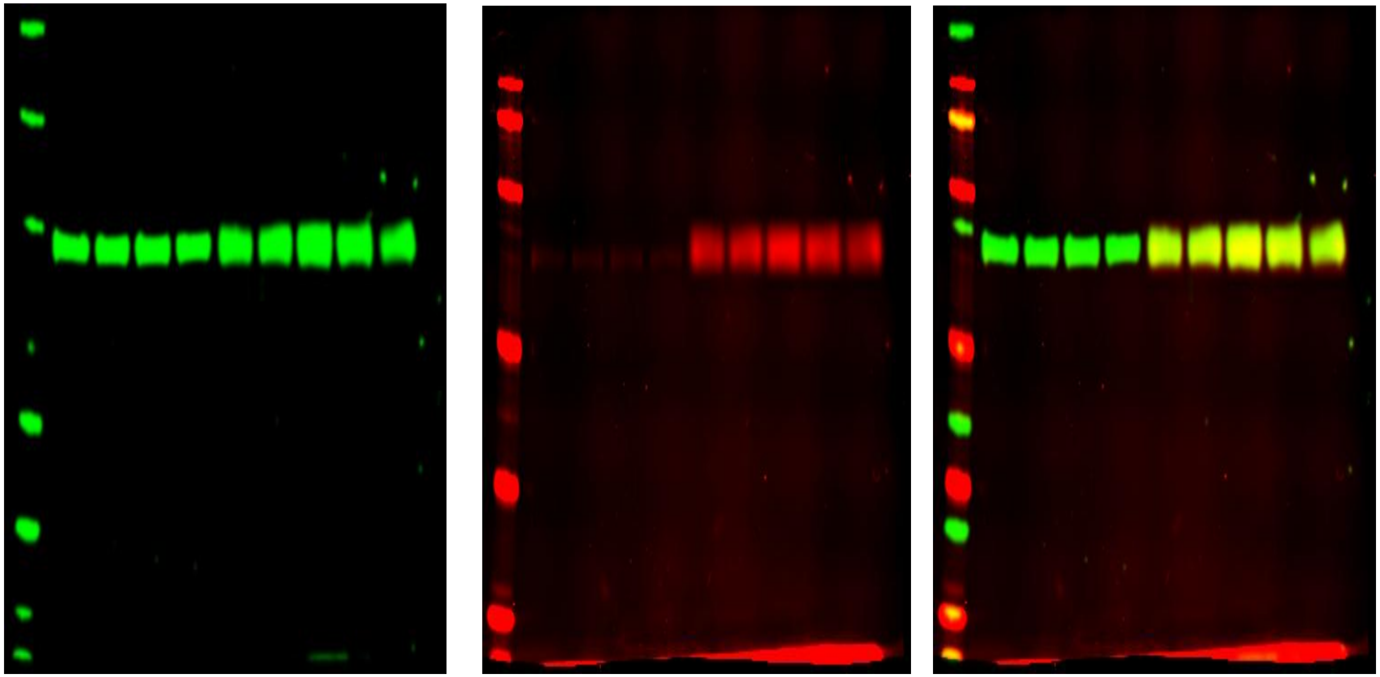

Supplemental Figure 8
